# Phyto-synthesized silver-iron oxide nanoparticles from *Traganum nudatum* for antimicrobial and anticancer applications: experimental and computational insights

**DOI:** 10.3389/fchem.2026.1877737

**Published:** 2026-09-09

**Authors:** Hessa O. Aldraiwiesh, Mohammed M. Elobeid, Zeyad E. Mohamed, Belal M. Kamel, Reham M. Aldahasi, Fatimah G. Albani, Nada K. Alharbi, Israa Salem, Hanan Henidi, Sahar S. Alghamdi, Mudawi M. Nour, Afrah E. Mohammed

**Affiliations:** 1 Department of Biology, College of Science, Princess Nourah Bint Abdulrahman University, Riyadh, Saudi Arabia; 2 Microbiology and Immunology Unit, Health Sciences Research Center, Princess Nourah Bint Abdulrahman University, Riyadh, Saudi Arabia; 3 College of Medicine, University Targu Mures Medical Campus Hamburg (UMCH), Hamburg, Germany; 4 College of Medicine, Cairo University, Cairo, Egypt; 5 College of Medicine, Assiut University, Assiut, Egypt; 6 Research Centre, King Faisal Specialist Hospital and Research Center, Riyadh, Saudi Arabia; 7 Research Department, Natural and Health Sciences Research Center, Princess Nourah Bint Abdulrahman University, Riyadh, Saudi Arabia; 8 Department of Pharmaceutical Sciences, College of Pharmacy, King Saud Bin Abdulaziz University for Health Sciences (KSAU-HS), Riyadh, Saudi Arabia; 9 King Abdullah International Medical Research Center (KAIMRC), Riyadh, Saudi Arabia; 10 King Abdulaziz Medical City, National Guard Health Affairs (NGHA), Riyadh, Saudi Arabia; 11 Habitat Regeneration and Landscaping Department, Wildlife and Natural Heritage, Royal Commission for AlUla, AlUla, Saudi Arabia

**Keywords:** anticancer, antimicrobial, *Candida albicans*, *Escherichia coli*, green synthesis, methicillin-resistant *Staphylococcus aureus* (MRSA)

## Abstract

The rising incidence of antimicrobial resistance necessitates the development of sustainable and eco-friendly nanomaterials. In this study, silver-iron oxide nanoparticles (Ag-FeO NPs) were phyto-synthesized using *Traganum nudatum* seed extract as a natural reducing and stabilizing agent, and their physicochemical properties and antimicrobial activity were systematically evaluated. UV-Vis spectroscopy showed a characteristic absorption peak at 359 nm, providing supportive evidence of nanoparticle formation. Fourier-transform infrared spectroscopy revealed functional groups at 1,645 and 3,315 cm^-1^, indicating the involvement of plant-derived phytochemicals in nanoparticle stabilization. Transmission electron microscopy analysis showed heterogeneous spherical and rod-like nanostructures with moderate aggregation, while scanning electron microscopy coupled with energy-dispersive X-ray spectroscopy qualitatively indicated the presence of silver and iron, together with oxygen and carbon. Dynamic light scattering analysis showed an average hydrodynamic particle size of 7.3 nm and a broad particle size distribution (PDI = 0.60), with a zeta potential of −1.36 mV. GC-MS profiling identified bioactive metabolites, including 3-(octanoyloxy)propane-1,2-diyl bis(decanoate), *n*-hexadecanoic acid, hentriacontane, and lidocaine, potentially contributing to nanoparticle synthesis and bioactivity. Ag-FeO NPs exhibited significant antimicrobial activity against *Escherichia coli*, methicillin-resistant *Staphylococcus aureus*, and *Candida albicans*. SEM analysis of *C. albicans* revealed severe ultrastructural damage, including surface deformation and cell wall disruption. Ag-FeO NPs produced greater reductions in MCF-7 cell viability than in MCF-10A non-tumorigenic epithelial cells, although a clear concentration-dependent response was not observed within the tested range. Computational prediction analyses using PASS, SwissTargetPrediction, SwissADME, and ProTox-II indicated that several identified phytochemicals possess antimicrobial and antineoplastic potential together with favorable pharmacokinetic characteristics. Overall, the integration of green synthesis, experimental validation, and computational modeling highlights the potential of *T. nudatum*-mediated Ag-FeO NPs as effective and sustainable agents for biomedical applications.

## 1 Introduction

In recent years, the field of nanotechnology has experienced remarkable growth. The foundation of nanotechnology is nanoparticles (NPs), which are microscopic particles with sizes ranging from 1 to 100 nm (Falke et al., 2024). The intrinsic qualities of nanoscale compounds, including optically active properties, reactivity, toughness, surface area, mechanical strength, and chemical reactivity, are what give them their significance (Sajid, 2022). NPs hold immense potential to transform numerous fields such as agriculture, medicine, and environmental sustainability (Atanda et al., 2025). The global nanotechnology market exceeds USD 100 billion and continues to grow rapidly, driven by applications in healthcare, agriculture, and environmental remediation. This expanding economic impact highlights the increasing demand for sustainable and eco-friendly nanoparticle synthesis approaches (Shaik et al., 2022). Due to their unique properties, which include electrical, optical, magnetic, and biocompatible properties, NPs are well-suited for medication administration and other medical applications (Zahin et al., 2020). Biocompatibility is essential for effective absorption of NPs, which can enter the body through the skin, circulation, lungs, blood-brain barrier, or stomach (Xuan et al., 2023). Depending on their size, shape, and structure, NPs have enhanced properties that are not always seen in bulk materials (Ghazal et al., 2024). In addition to size and composition, nanoparticle morphology plays a critical role in determining their physicochemical and biological properties. Nanoparticles can be engineered into various shapes, including spherical, cubic, rod-shaped, and nanoflower-like structures, each exhibiting distinct surface characteristics, reactivity, cellular interactions, and catalytic performance. Morphological control has therefore emerged as an important strategy for tailoring nanoparticle functionality and optimizing their applications in biomedical, agricultural, and environmental fields (Theodosiou et al., 2022; Wang et al., 2020). Furthermore, NPs can be synthesized using metal, carbon, metal oxides, or organic materials (Dubey et al., 2024). Particularly, the scientific world has taken notice of silver nanoparticles (AgNPs) because of their broad-spectrum antibacterial action and prospective usage in other medicinal sectors (Luceri et al., 2023). Furthermore, modern green nanotechnology is often interested in iron oxide nanoparticles (FeO NPs) because of their excellent catalytic and antibacterial capabilities, high magnetic susceptibility, and super-paramagnetic characteristics (Ezealigo et al., 2021). Bimetallic compounds are composed of two metallic elements. The purposeful synthesis of bimetallic complexes has gained popularity due to their numerous adjustable characteristics, including the polarity and sequence of metal-metal bonds (Campos, 2020). The distinct mixing patterns and synergistic effects of two metals make the bimetallic nanoparticles more attractive than monometallic nanoparticles (Dlamini et al., 2023). Bimetallic nanoparticles (BNPs) are nanoscale materials composed of two different metallic elements (Liu and Corma, 2023). Because of their synergistic qualities, they may be used in a variety of fields, including biological applications (Idris and Roy, 2023). Anticancer medicines and photosynthesizers, which are useful in treating cancer, can be delivered via the BNPs in a straightforward, adaptable, and accessible manner to users (Makada et al., 2023). In comparison to these conventional methods, a greener path of NP synthesis offers an economical, environment-friendly, and nontoxic approach (Bhardwaj et al., 2020). Although BNPs have shown great results on their own in various fields, the addition of plant extracts, due to their natural reducing and stabilizing properties, has boosted the properties of these nanoparticles considerably (Pirsaheb et al., 2024). A significant advantage of employing plant extracts in the manufacture of nanoparticles is the use of natural preserving and minimizing agents, which eliminates the need for highly toxic substances and increases biocompatibility (Villagrán et al., 2024). Research indicates that plant extracts can effectively synthesize BNPs, enhancing their catalytic, antibacterial activities (Al-Haddad et al., 2020), anticancer efficacy (Kalakonda et al., 2024), and providing sustainable solutions for clean water (Kalakonda et al., 2024). In the environmentally friendly manufacture of nanoparticles, secondary metabolites, including terpenes, alkaloids, flavonoids, and phenols, which are produced by plants as defense mechanisms against pathogens, can function as stabilizing and reducing agents (Barathikannan et al., 2023). A variety of plant extracts have been employed in the green synthesis of bimetallic nanoparticles, such as *Pulicaria undulata* Extract (Khan et al., 2020), leaf extract of *Salvia officinalis* (Malik et al., 2023), and clove buds extract (Sharma et al., 2020).

The herbaceous wild plant *Traganum nudatum,* which belongs to the *Chenopodiaceae* family, is extensively utilized in Algerian traditional medicine as a therapeutic herb (Mouderas et al., 2019). The methanol and chloroform extracts of *T. nudatum,* in particular, are suspected to have anti-inflammatory, antidiabetic, and anticancer effects (El-bassossy, 2022). Water extracts from the roots, stems, and leaves of *T*. *nudatum* have demonstrated antibacterial activity against five clinical bacterial strains, including *Escherichia coli*, *Staphylococcus aureus*, *Streptococcus* sp., *Salmonella typhi*, and *Pseudomonas aeruginosa*. The extracts showed greater efficacy against Gram-positive bacteria, with root extracts exhibiting the strongest antibacterial activity (Labed et al., 2010). Moreover, *T. nudatum* ethanolic extracts show strong anti-hyperglycemic potential and may be more successful than the common medication Glibenclamide in treating the pathophysiology of diabetes (Badawneh et al., 2017). The biogenic agent *Traganum nudatum* was employed in this study for the green synthesis of bimetallic silver-iron oxide (Ag-FeO) nanoparticles, with a strong emphasis on sustainability. To the best of our knowledge, there are currently no reputable published studies reporting the use of *T. nudatum* in nanoparticle synthesis. This research explores, for the first time, the potential of *T. nudatum* as a novel biological resource for the development of environmentally friendly and sustainable nanomaterials.

## 2 Materials and methods

### 2.1 Materials

*T. nudatum* seeds were obtained from the Royal Commission for AlUla nursery, AlUla, Saudi Arabia. Silver nitrate (AgNO_3_, ≥99.9%, Sigma-Aldrich, USA), ferric chloride hexahydrate (FeCl_3_·6H_2_O, ≥98%, Sigma-Aldrich, USA), and ferrous chloride tetrahydrate (FeCl_2_·4H_2_O, ≥99%, Sigma-Aldrich, USA) were used as metal precursors. All chemicals and reagents were obtained from the Laboratory of Health Sciences Research at Princess Nourah bint Abdulrahman University.

### 2.2 The preparation of aqueous extracts

*T. nudatum* seeds were thoroughly washed with distilled water to remove dust and surface impurities, then dried overnight at 70 °C and milled into a fine powder. For preparation of the aqueous extract, 2.5 g of the powdered seeds was mixed with 50 mL of distilled water and heated in a water bath at 90 °C for 15 min. The resulting extract was allowed to cool and subsequently filtered using filter paper with a pore size of 20 µm to remove particulate matter. The filtrate was then used for further experimental procedures (Mohammed et al., 2026).

### 2.3 Synthesis of bimetallic nanoparticles

For nanoparticle synthesis, AgNO_3_ (1 mM), FeCl_3_·6H_2_O (10 mM), and FeCl_2_·4H_2_O (5 mM) were used. A 5 mL of the prepared seed extract was added to a reaction flask containing 10 mL of iron oxide precursor solution and 10 mL of silver precursor solution. The reaction mixture was heated in a water bath at 90 °C for 15 min. A noticeable change in both color and solution density indicated the formation of nanoparticles. After cooling to room temperature, the mixture was centrifuged at 5,500 × g for 30 min to collect the synthesized nanoparticles (Aldahasi and Mohammed, 2025). A schematic representation of the green synthesis methods is shown in Figure 1.

**FIGURE 1:**
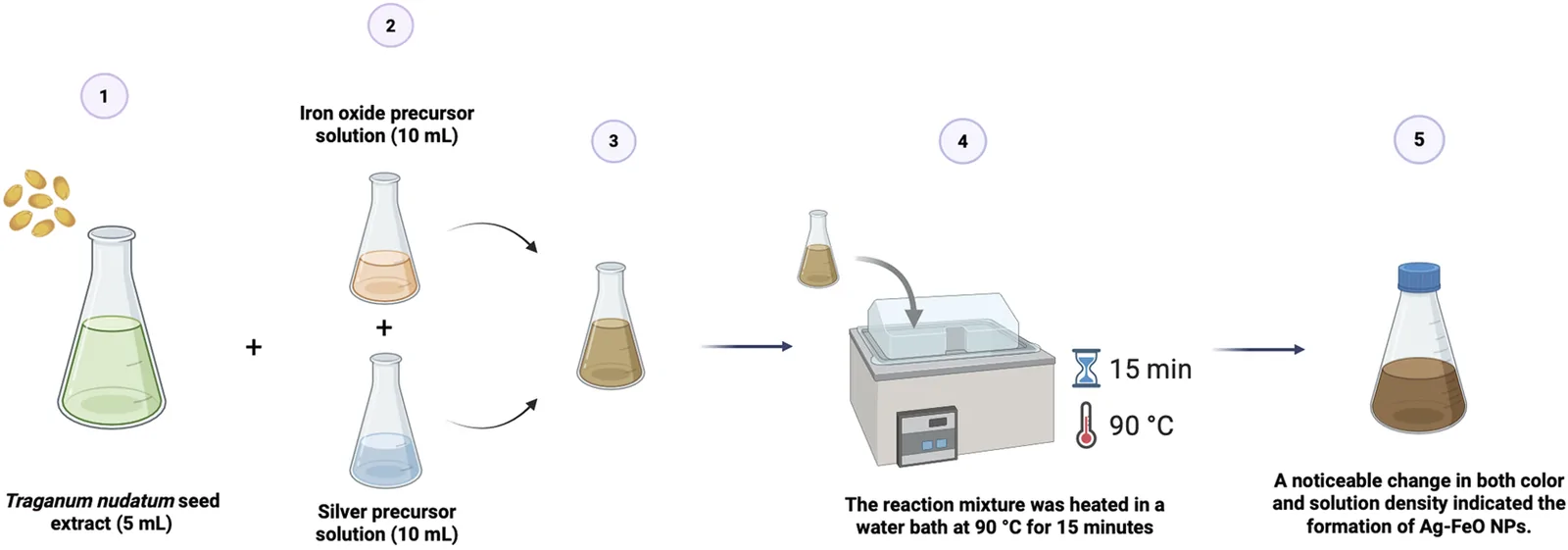
Schematic representation of the green synthesis of Ag-FeO nanoparticles (Ag-FeO NPs) using *Traganum nudatum* seed extract.

### 2.4 Characterization of Ag-FeO NPs

#### 2.4.1 Ultraviolet-visible spectroscopy

Thermo Fisher Scientific, based in Waltham, Massachusetts, USA, provided the Evolution 201 UV-visible spectrophotometer, which was used to analyze UV-Vis spectra. After 24 h, using distilled water as a reference, the reaction mixture was analyzed three times across a wavelength range of 200–500 nm.

#### 2.4.2 Scanning electron microscopy (SEM) and transmission electron microscopy (TEM)

The morphology and elemental composition of the synthesized Ag-FeO NPs were characterized using a field-emission scanning electron microscope (JSM-IT500HR; JEOL Ltd., Tokyo, Japan) equipped with energy-dispersive X-ray spectroscopy (EDX; STD-PC80 detector) operating at an accelerating voltage of 15 kV. Before imaging, liquid samples (100–300 μL) were deposited onto carbon tape, air-dried overnight, and coated with platinum using a JEC-3000FC auto fine coater (JEOL Ltd., Tokyo, Japan). The particle size and morphology were further examined using a transmission electron microscope (JEM-1400Flash; JEOL Ltd., Tokyo, Japan) operated at an accelerating voltage of 120 kV. Samples were suspended in 70% ethanol, sonicated for 15–20 min, deposited (10 μL) onto carbon-coated nickel grids, and analyzed under TEM.

#### 2.4.3 Dynamic light scattering (DLS) and zeta potential

Hydrodynamic particle size distribution, PDI, and zeta potential measurements of Ag-FeO NPs were carried out using Zetasizer (NANO ZSP; Malvern Instruments Ltd., Serial Number: MAL1118778, ver 7.11, Malvern, UK).

#### 2.4.4 Fourier-transform infrared spectroscopy

FTIR analysis was performed on both the *T. nudatum* seed extract and the synthesized Ag-FeO NPs to identify functional groups involved in nanoparticle formation and stabilization using FTIR spectroscopy (SPECTRUM100; Perkin-Elmer, Waltham, MA, USA), which has a scanning range of 500 to 4,000 cm^-1^.

### 2.5 Gas chromatography-mass spectrometry (GC-MS)

For GC-MS analysis, 5 g of dried *Traganum nudatum* seed powder was macerated in 100 mL of ethanol under continuous shaking at room temperature for 24 h. The resulting extract was first filtered through 20 μm filter paper to remove insoluble plant debris and subsequently passed through a 0.22 μm syringe filter. A 1 μL aliquot of the clarified extract was then analyzed using an Agilent 7890B gas chromatograph coupled to an Agilent 7000D mass spectrometer (Agilent Technologies, Santa Clara, CA, USA).

Compound identification was achieved by matching the obtained mass spectra with those available in the National Institute of Standards and Technology (NIST 17) Mass Spectral Library and published reference spectra. The relative abundance of the identified constituents was determined from the percentage peak area of each chromatographic peak in the total ion chromatogram (TIC), without applying response correction factors. The compounds were subsequently listed according to their relative peak area (%).

### 2.6 *In vitro* antimicrobial assay

Using *Candida albicans (*ATCC 10231), *Escherichia coli* (ATCC 25922)*,* and methicillin-resistant *Staphylococcus aureus* (ATCC 43300), the antimicrobial susceptibility assay was carried out using the agar well diffusion assay (Akinsiku et al., 2018) as an initial qualitative screening assay method to assess antibacterial susceptibility. The microorganisms were examined for their susceptibility to the bio-fabricated BNPs and the seeds extract. Microbial suspensions were produced in saline at 0.5 McFarland, approximately 1.5 × 10^8^ CFU (Colony-forming units)/mL). Wells were created in agar plates inoculated with each microbial strain. After the plates had dried, 40 μL of Ag-FeO NPs (40 mg/mL) and seed extract (2 g/100 mL) were added individually in a sterile setting.

The area surrounding each well (zones of inhibition) was then measured after the agar plates were incubated for 24 h at 37 °C. Ampicillin and fluconazole were used as positive controls against bacteria and *C. albicans*, respectively, and sterile water was used as a negative control. Because *Candida albicans* exhibited the greatest susceptibility to the biosynthesized Ag-FeO NPs among the tested microorganisms, it was selected for SEM analysis to investigate nanoparticle-induced morphological alterations. Treatments were performed in triplicate.

### 2.7 SEM evaluation of morphological changes in *Candida albicans* following Ag-FeO nanoparticle treatment

For SEM analysis, three independent biological replicates were performed. *C. albicans* cells were exposed to Ag-FeO NPs (40 mg/mL) for 24 h at 37 °C prior to fixation and SEM analysis (Akinremi et al., 2018; Hussein-Al-Ali et al., 2014). *C. albicans* cells were collected from overnight cultures and rinsed with phosphate buffer to eliminate residual growth medium. Gram-stained *C. albicans* smears were allowed to air-dry and were mounted on aluminium stubs using conductive carbon tape, and the samples were sputter-coated with platinum for 50 s at 20 µA (JEC-3000FC, JEOL, Japan). Imaging was performed at an accelerating voltage of 15 kV using a field-emission scanning electron microscope (JSM-IT500HR, JEOL, Japan). SEM was used to provide complementary visualization of *C. albicans* distribution and cellular morphology within the stained smears.

### 2.8 Cytotoxicity

The cytotoxic effects of Ag-FeO NPs on MCF-7 (human breast adenocarcinoma cell line) and the control cell line MCF-10A (non-tumorigenic epithelial cell line) were evaluated according to Ahmad et al. (2023) with some modifications. Both cell lines were obtained from the Health Sciences Research Center Core Lab at Princess Nourah University. Cell viability was assessed using the MTT assay. Cells were seeded in 96-well plates at 5 × 10^4^ cells/well and allowed to adhere overnight at 37 °C with 5% CO_2_. On the second day, cells were exposed to varying concentrations of Ag-FeO NPs, ranging from 15 to 500 μg/mL, and incubated for 48 h. After treatment, the medium was replaced with fresh medium containing 3-(4, 5-dimethylthiazol-2-yl)-2,5-diphenyltetrazolium bromide (MTT, 0.5 mg/mL), and cells were incubated for 3–4 h at 37 °C. The resulting formazan crystals were dissolved in dimethyl sulfoxide, and absorbance was measured at 570 nm using a BioTek Synergy HTX microplate reader (BioTek Instruments, Winooski, VT, USA). All treatments were performed in triplicate.

### 2.9 In silico computational analysis

#### 2.9.1 Activity prediction

The active compounds were prepared by generating Simplified Molecular Input Line Entry System (SMILES) and obtaining the two-dimensional (2D) chemical structures from the ChemDraw 12 Pro, followed by computational prediction of their anticancer and antimicrobial activities (antibacterial and antifungal) by utilizing the online service Prediction of Activity Spectra for Substance (PASS) (https://www.way2drug.com/PassOnline/index.php).

This tool predicts the biological activities of compounds based on models derived from known bioactive ligands. For each predicted activity, it reports two probability values: the probability of activity (Pa) and the probability of inactivity (Pi) (Filimonov et al., 2014).

#### 2.9.2 Target prediction

To explore the targets for identified compounds of *Traganum nudatum* seed extract, a target prediction was performed using the SwissTargetPrediction online website (http://www.swisstargetprediction.ch/) that predicts the targets of bioactive compounds based on 2D and 3D similarity measures with known ligands. Simplified Molecular Input Line Entry System (SMILES) strings were used as input to the SwissTargetPrediction platform to predict potential protein targets (Gfeller et al., 2014).

#### 2.9.3 Absorption, distribution, metabolism, and excretion (ADME) prediction

The calculations of the absorption, distribution, metabolism, and excretion (ADME) properties of the *Traganum nudatum* seed extract compounds were performed using the SwissADME website (http://www.swissadme.ch/), a website developed and maintained by the Molecular Modeling Group of the Swiss Institute of Bioinformatics (SIB). The SMILES of each compound were utilized to generate data from ADME. This platform serves as a valuable resource for evaluating the pharmacokinetic and medicinal chemistry characteristics of molecules, thereby facilitating informed decision-making in drug design and development (Daina et al., 2017).

#### 2.9.4 Cytochromes P450 (CYP) enzyme inhibition profile

The SwissADME website was also used for predicting the cytochrome P450 enzyme inhibition profile for each *Traganum nudatum* seed extract compound (http://www.swissadme.ch/). The effect of the identified compounds was predicted on five important cytochrome P450 enzymes, including CYP1A2, CYP2C19, CYP2C9, CYP2D6, and CYP3A4 (Daina et al., 2017).

#### 2.9.5 Organ toxicity and safety profile predictions

The ProTox-II platform was used (https://tox.charite.de/protox3/#) to predict the potential toxicity of the compounds that are identified in *Traganum nudatum* seed extract. Numerous types of toxicity were evaluated, including hepatotoxicity, carcinogenicity, cytotoxicity, mutagenicity, and immunotoxicity for the compounds, by using SMILES as an input to generate the predictions (Banerjee et al., 2018).

### 2.10 Statistical analysis

Data are presented as mean ± standard deviation. Statistical analyses were performed using GraphPad Prism 9.1 (GraphPad Software Inc., La Jolla, CA, USA). One-way ANOVA was used to evaluate treatment effects, followed by Tukey’s multiple comparisons test to evaluate differences in antimicrobial activity (inhibition zone diameters) among treatments. Nonlinear regression analysis was applied to determine half maximal inhibitory concentration (IC_50_) values. For the cytotoxicity assay, cell viability data were analyzed using two-way ANOVA to evaluate the effects of cell type, nanoparticle concentration, and their interaction, followed by Šídák’s multiple comparisons test to compare MCF-7 and MCF-10A cells at each concentration. In addition, one-way ANOVA was performed separately within each cell line to assess concentration-dependent effects. Statistical significance was set at P < 0.05. Particle size distribution was determined from TEM images using ImageJ software, while FTIR and UV-Vis spectra were processed using OriginPro 2023b (OriginLab Corporation, Northampton, MA, USA).

## 3 Results

### 3.1 Ultraviolet-visible spectroscopy

The UV-Vis absorption spectrum of the synthesized Ag-FeO NP suspension exhibited a maximum absorbance peak at 359 nm, as shown in Figure 2. Although comparative spectra of the *T. nudatum* seed extract and the individual metal precursor solutions were not available, the observed spectral feature, when considered together with the complementary characterization results, provides supportive evidence of nanoparticle formation.

**FIGURE 2:**
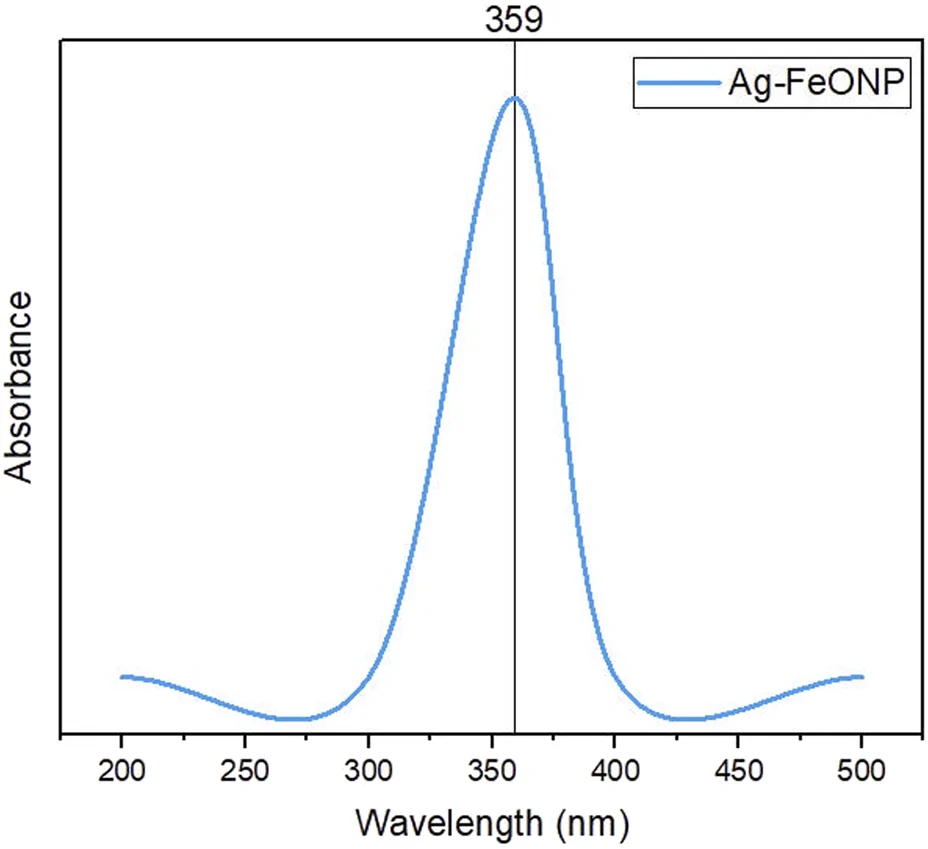
UV-Vis absorption spectrum of phyto-synthesized Ag-FeO NPs produced using *Traganum nudatum* seed extract. An absorption maximum was observed at 359 nm. The spectrum shown is representative of three independent synthesis experiments (n = 3).

### 3.2 Scanning electron microscopy (SEM) and transmission electron microscopy (TEM)

Transmission electron microscopy (TEM) analysis revealed that the synthesized Ag-FeO NPs predominantly exhibited rod-like morphologies, while some particles appeared more spherical. A degree of particle agglomeration was also observed, which limited precise shape differentiation in certain regions, as shown in Figure 3A.

**FIGURE 3:**
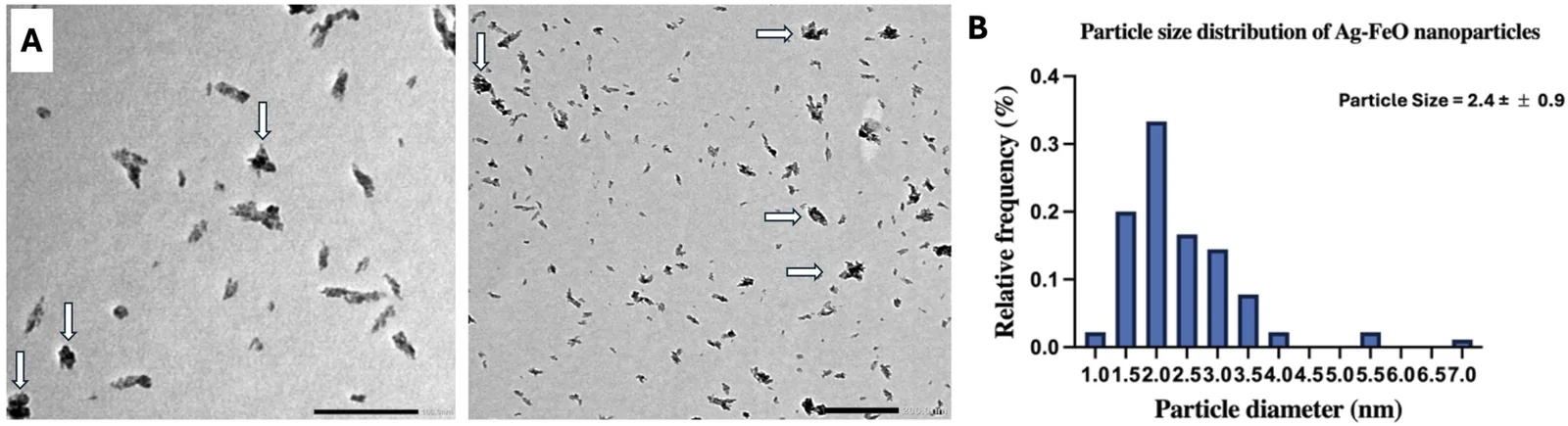
Transmission electron microscopy (TEM) characterization of phyto-synthesized Ag-FeO NPs. **(A)** Representative TEM micrographs at different magnifications showing heterogeneous nanoparticle morphologies, including predominantly spherical and rod-like particles with moderate agglomeration. White arrows indicate representative agglomerated nanoparticle clusters. Scale bars represent 100 nm (left) and 200 nm (right). **(B)** Particle size distribution histogram generated from TEM images using ImageJ software based on measurements of 90 individual nanoparticles.

Particle size distribution analysis was performed using ImageJ software based on measurements of 90 individual nanoparticles from TEM micrographs. The Ag-FeO NPs exhibited a relatively narrow size distribution with a mean particle size of 2.4 ± 0.9 nm, with most particles ranging between approximately 1.5 and 3.5 nm and a maximum observed diameter of approximately 6.8 nm. The particle size distribution histogram is presented in Figure 3B.

SEM micrographs (Figure 4A) revealed a heterogeneous surface composed of aggregated nanoparticle clusters. Due to the extensive agglomeration, the morphology of individual Ag-FeO NPs could not be clearly distinguished. Figure 4B shows the SEM image indicating the region selected for EDX analysis (Spc_002). EDX analysis (Figure 4C) qualitatively indicated the presence of Ag and Fe in the synthesized material. Characteristic Ag signals were observed at approximately 2.98 keV, while Fe signals appeared at approximately 0.7 and 6.4 keV. Oxygen and carbon signals were also detected and may be associated with iron oxide and plant-derived organic constituents, respectively. However, quantitative EDX measurements, including weight percentage (wt%) and atomic percentage (at%), were not available; therefore, the relative elemental composition and purity of the synthesized material could not be determined from the present EDX analysis.

**FIGURE 4:**
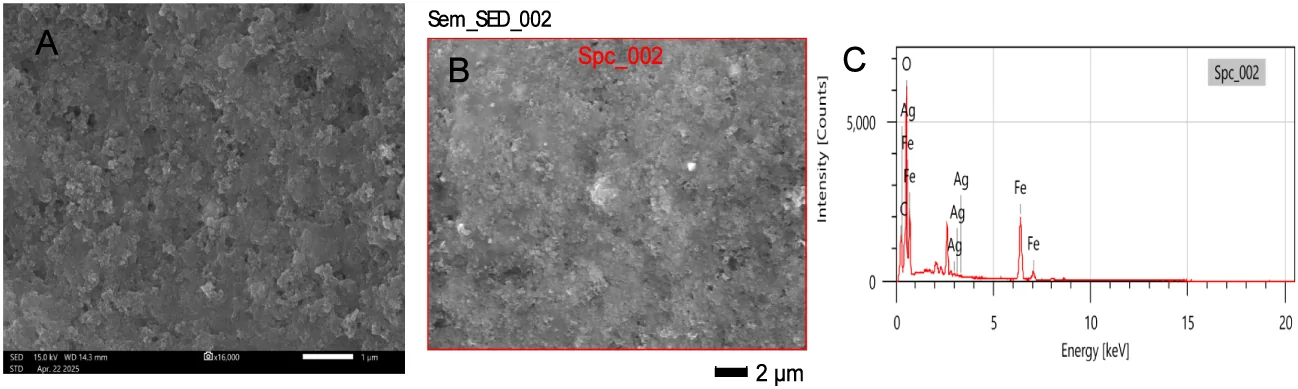
Scanning electron microscopy (SEM) and energy-dispersive X-ray spectroscopy (EDX) characterization of the phyto-synthesized Ag-FeO NPs. **(A)** Representative SEM micrograph showing the morphology of the biosynthesized Ag-FeO NPs, with irregularly shaped aggregated nanoparticle clusters. **(B)** SEM image indicating the region selected for EDX analysis (Spc_002). **(C)** EDX spectrum showing signals corresponding to Ag, Fe, O, and C. The Ag and Fe signals qualitatively support the presence of both metallic components in the synthesized material, while O and C may be associated with iron oxide and plant-derived organic constituents, respectively. Because quantitative EDX data (wt% and at%) were not available, the relative elemental composition and purity could not be determined by EDX.

### 3.3 Dynamic light scattering (DLS) and zeta potential

Polydispersity index (PDI), surface zeta potential, and hydrodynamic particle size of the synthesized Ag-FeO NPs were determined using dynamic light scattering (DLS), as shown in Figure 5. The Ag-FeO NPs exhibited an average hydrodynamic particle size of 7.3 nm, a PDI value of 0.60, and a zeta potential of −1.36 mV. The particle size distribution showed a major peak corresponding to the primary nanoparticle population, along with minor peaks below 1 d.nm and in the 10^3^–10^4^ d.nm region. The DLS analysis therefore revealed a broad particle size distribution within the nanoparticle suspension.

**FIGURE 5:**
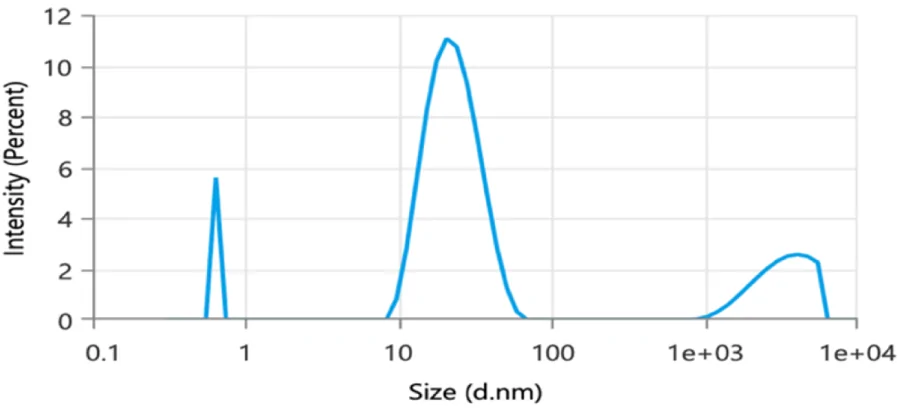
Hydrodynamic particle size distribution of phyto-synthesized Ag-FeO NPs prepared using *Traganum nudatum* seed extract, determined by dynamic light scattering (DLS). The presented distribution is representative of three independent measurements (n = 3).

### 3.4 Phytochemical characterization of *Traganum nudatum* seed extract and Ag-FeO NPs

Fourier-transform infrared (FTIR) spectroscopy and gas chromatography-mass spectrometry (GC-MS) analyses were performed to characterize the phytochemical constituents of the *Traganum nudatum* seed extract and to identify compounds potentially involved in nanoparticle synthesis and biological activity.

The FTIR spectra of both the *T. nudatum* seed extract and the synthesized Ag-FeO NPs are presented in Figure 6. Characteristic absorption bands were observed at 3,315 and 1,645 cm^-1^, corresponding to O-H/N-H and C=O stretching vibrations, respectively. The presence of these bands in both spectra suggests that plant-derived phytochemicals remained associated with the Ag-FeO NPs surface and likely contributed to nanoparticle reduction and stabilization.

**FIGURE 6:**
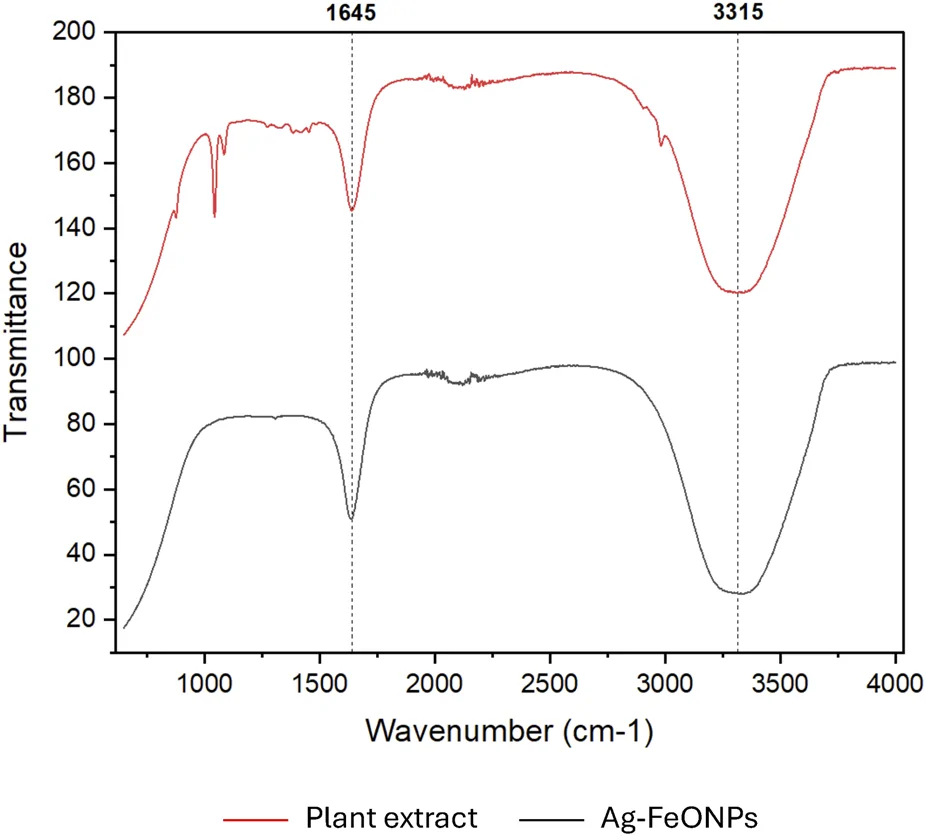
FTIR spectra of *Traganum nudatum* seed extract (red spectrum) and biosynthesized Ag-FeO NPs (black spectrum). Characteristic absorption bands at 3,315 cm^-1^ and 1,645 cm^-1^ correspond to O-H/N-H stretching and C=O stretching vibrations, respectively, indicating the involvement of plant-derived phytochemicals in the reduction and stabilization of Ag-FeO NPs.

To further investigate the chemical composition of the seed extract, GC-MS analysis was performed. Approximately 97 compounds were identified by GC-MS analysis. Based on relative abundance and peak intensity, the eight major compounds were selected for subsequent *in silico* analyses, including PASS, SwissTargetPrediction, SwissADME, and ProTox-II evaluations. Selected compounds were 3-(octanoyloxy)propane-1,2-diyl bis(decanoate), carbonic acid eicosyl vinyl ester, benzyldiethyl-(2,6-xylylcarbamoylmethyl) ammonium benzoate, sebacic acid di (2-octyl) ester, 2-methylhexacosane, hentriacontane, lidocaine, and n-hexadecanoic acid (Figure 7; Table 1). These identified compounds were subsequently subjected to computational analyses to predict their biological activities, pharmacokinetic properties, and toxicity profiles. Although post-synthesis GC-MS analysis was not performed, FTIR spectra indicated the persistence of plant-derived functional groups associated with the Ag-FeO NPs, supporting the potential involvement of phytochemicals in nanoparticle reduction and stabilization.

**FIGURE 7:**
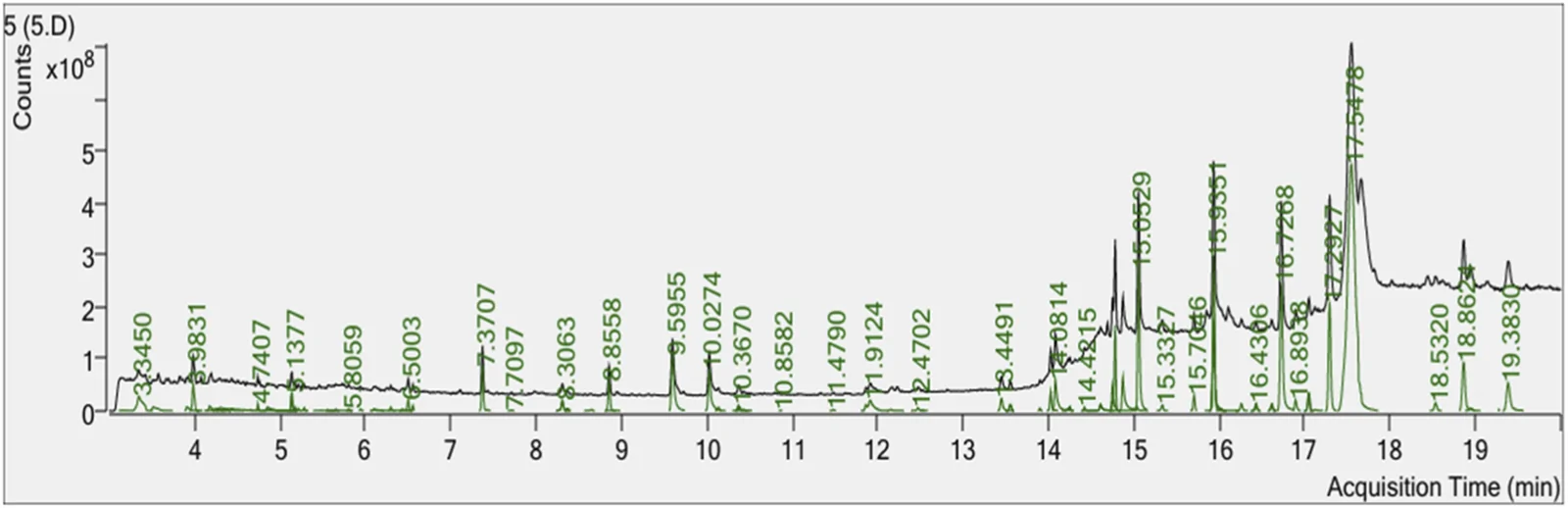
Gas chromatography-mass spectrometry (GC-MS) total ion chromatogram (TIC) of the ethanolic seed extract of *Traganum nudatum*. The chromatogram shows the separation of the detected phytochemical constituents according to their retention times. Compound identification was performed by comparing the acquired mass spectra with the NIST 17 Mass Spectral Library. Peak numbers correspond to the identified compounds listed in Table 1.

**TABLE 1:** The most abundant compounds from the *Traganum nudatum* seed extract by GC-MS analysis.

No.	Component retention time (RT, min)	Compound name	Formula
1	17.5478	3-(Octanoyloxy) propane-1,2-diyl bis(decanoate)	C_31_H_58_O_6_
2	15.9351	Carbonic acid, eicosyl vinyl ester	C_23_H_44_O_3_
3	15.0529	Benzyldiethyl-(2,6-xylylcarbamoylmethyl) ammonium benzoate	C_28_H_34_N_2_O_3_
4	16.7268	Sebacic acid, di (2-octyl) ester	C_26_H_50_O_4_
5	17.2927	2-Methylhexacosane	C_27_H_56_
6	18.8624	Hentriacontane	C_31_H_64_
7	9.5955	Lidocaine	C_14_H_22_N_2_O
8	10.0274	*n*-Hexadecanoic acid	C_16_H_32_O_2_

Therefore, the *in silico* analyses should be considered supportive evidence of the potential biological activities of the extract constituents rather than direct proof of their presence in the final nanoparticle formulation.

### 3.5 Antimicrobial activity

The antimicrobial activity of the synthesized Ag-FeO NPs was evaluated against selected pathogenic microorganisms, including *E. coli*, methicillin-resistant *Staphylococcus aureus* (MRSA), and *C. albicans*. The nanoparticles exhibited inhibitory activity against all tested microorganisms, producing inhibition zones of 10.9 ± 0.5 mm, 12.8 ± 0.8 mm, and 17.6 ± 1.2 mm against *E. coli*, MRSA, and *C. albicans*, respectively (Figure 8). The antimicrobial efficacy of Ag-FeO NPs was further compared with conventional antimicrobial agents. Against *E. coli*, the inhibition zone produced by Ag-FeO NPs was slightly lower than that of ampicillin. In contrast, Ag-FeO NPs exhibited greater inhibitory activity against MRSA than ampicillin, whereas fluconazole produced a larger inhibition zone against *C. albicans* than the nanoparticles. These findings demonstrate the antimicrobial potential of Ag-FeO NPs against both bacterial and fungal pathogens. No activity was noted for the seed extract and the sterile water.

**FIGURE 8:**
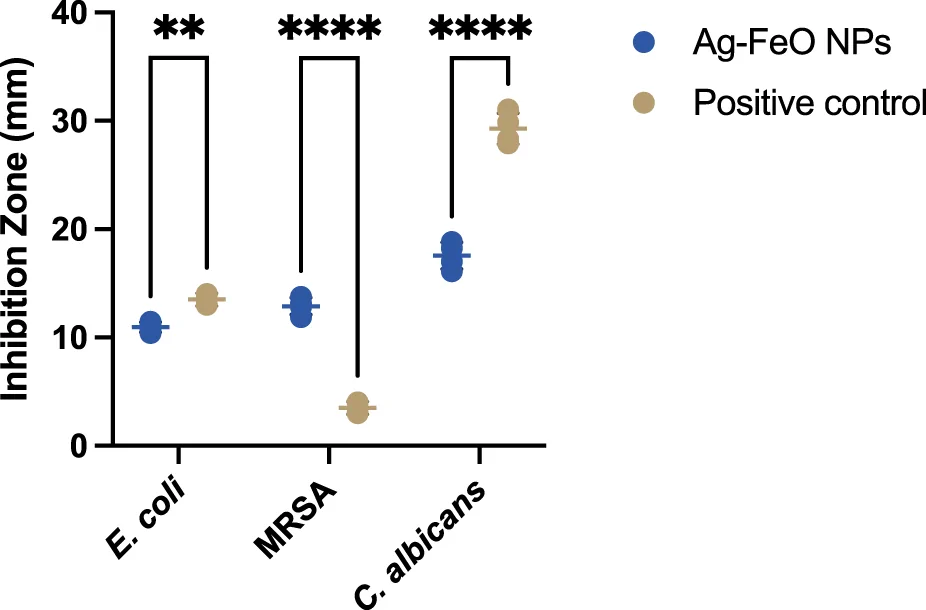
Antimicrobial activity of Ag-FeO NPs (40 mg/mL) against *E. coli*, methicillin-resistant *Staphylococcus aureus* (MRSA), and *Candida albicans*, expressed as inhibition zone diameter (mm). Ag-FeO NP activity was compared with conventional antimicrobial controls, including ampicillin for bacterial strains and fluconazole for *C. albicans*. Asterisks denote significant differences between treatments (**P < 0.01, ****P < 0.0001). Data are presented as mean ± SE from three independent biological replicates (n = 3).

### 3.6 SEM micrograph of *Candida albicans*

The control samples displayed the typical ovoid morphology of *C. albicans*, characterized by smooth, intact cell surfaces with no observable structural alterations, as shown in Figure 9A. In contrast, *C. albicans* cells treated with 40 mg/mL Ag-FeO NPs exhibited pronounced surface morphological changes (Figures 9B–D). The treated cells showed rough, uneven surfaces with a granular texture, likely resulting from nanoparticle adherence. Several cells demonstrated marked deformation, including surface pitting and indentations. In addition, filamentous structures resembling hyphae or pseudohyphae were observed in multiple fields, suggesting a stress-induced response and altered growth behavior. Overall, the treated cells appeared aggregated, irregular in shape, and partially elongated, with evident clumping. The transition from smooth to rough cell surface ultrastructure indicates substantial damage to the outer cell wall.

**FIGURE 9:**
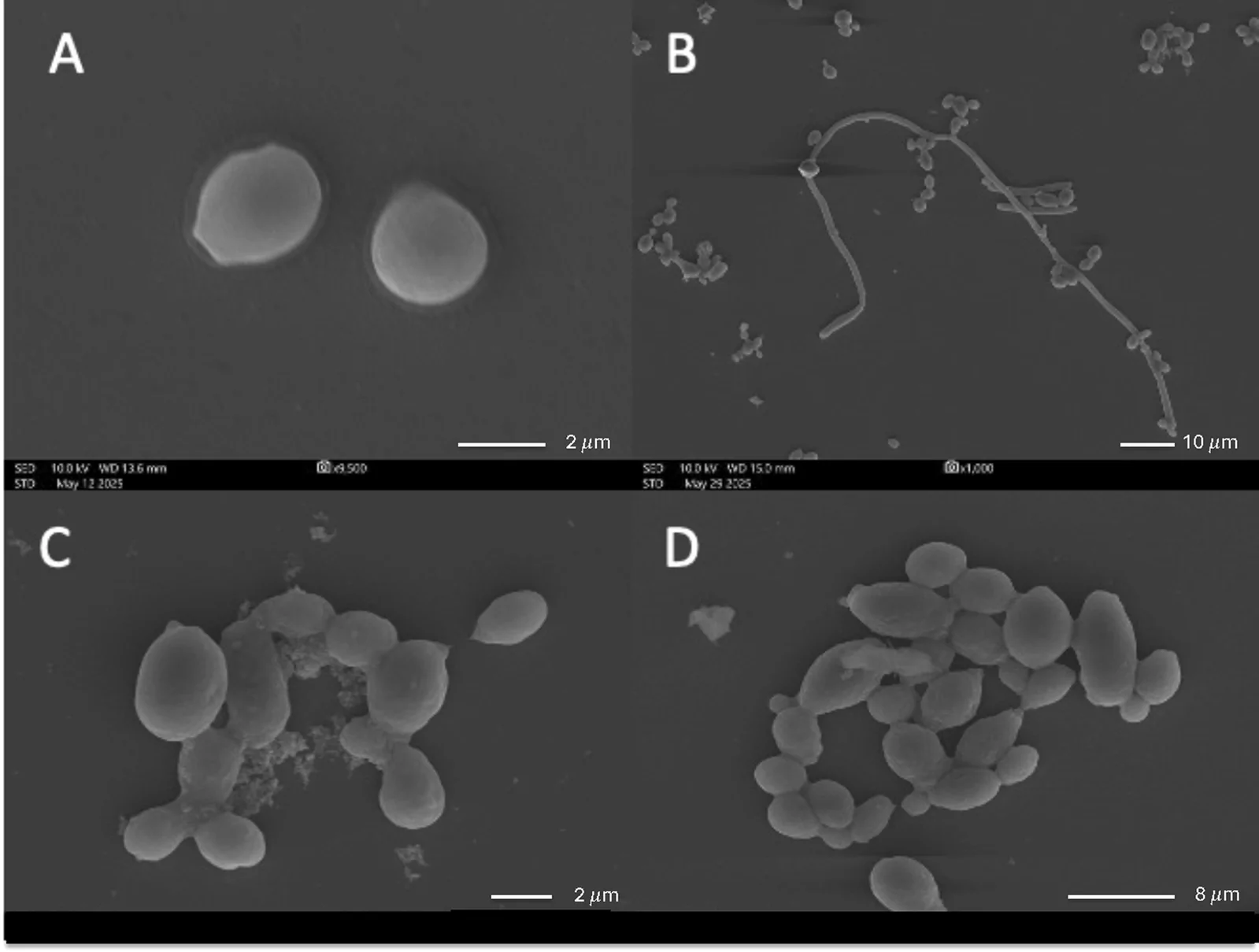
Scanning electron microscopy (SEM) images of *Candida albicans*. **(A)** Untreated control cells showing typical smooth, ovoid morphology (×9,500 magnification). **(B–D)** *C. albicans* cells treated with 40 mg/mL Ag-FeO NPs, exhibiting pronounced morphological alterations at magnifications of ×1,000 **(B)**, 6,000× **(C)**, and 4,300× **(D)**, including surface roughening, deformation, aggregation, and evidence of nanoparticle adherence.

### 3.7 Cytotoxic effects of Ag-FeO NPs on MCF-7 and MCF-10A cells

The cytotoxic effects of Ag-FeO NPs on MCF-7 breast cancer cells and MCF-10A non-tumorigenic epithelial cells were evaluated using the MTT assay (Figure 10). MCF-7 cells exhibited moderate reductions in viability, ranging from 73.56% to 87.06% across the tested concentrations (15–500 μg/mL). In contrast, MCF-10A cells maintained higher viability values, ranging from 84.59% to 97.96%, indicating lower susceptibility to Ag-FeO NP exposure. Two-way ANOVA revealed a significant effect of cell type on viability (P = 0.0022), whereas neither concentration (P = 0.7578) nor the interaction between concentration and cell type (P = 0.4450) was significant. Pairwise comparisons using Šídák’s multiple comparisons test showed a significant difference between MCF-7 and MCF-10A cells only at 500 μg/mL (P = 0.0447). Furthermore, one-way ANOVA performed separately for each cell line demonstrated no significant concentration-dependent effect on MCF-7 (P = 0.4148) or MCF-10A (P = 0.7260) viability. Nonlinear regression analysis estimated IC_50_ values of 818.5 μg/mL for MCF-7 cells and 5,106 μg/mL for MCF-10A cells. However, the dose-response curves showed poor goodness-of-fit to the sigmoidal model (*R* ^2^ = −8.203 for MCF-7 and -4.399 for MCF-10A), indicating that the calculated IC_50_ values should be interpreted with caution. Overall, the findings suggest that Ag-FeO NPs had greater cytotoxic effects on MCF-7 cells than on MCF-10A cells, although the observed responses did not exhibit a clear concentration-dependent trend within the tested concentration range. Because the *T. nudatum* seed extract was not evaluated independently in the MTT assay, the present results do not establish whether the observed cytotoxic effects are attributable specifically to the Ag-FeO NP formulation, the associated plant-derived constituents, or their combined effects.

**FIGURE 10:**
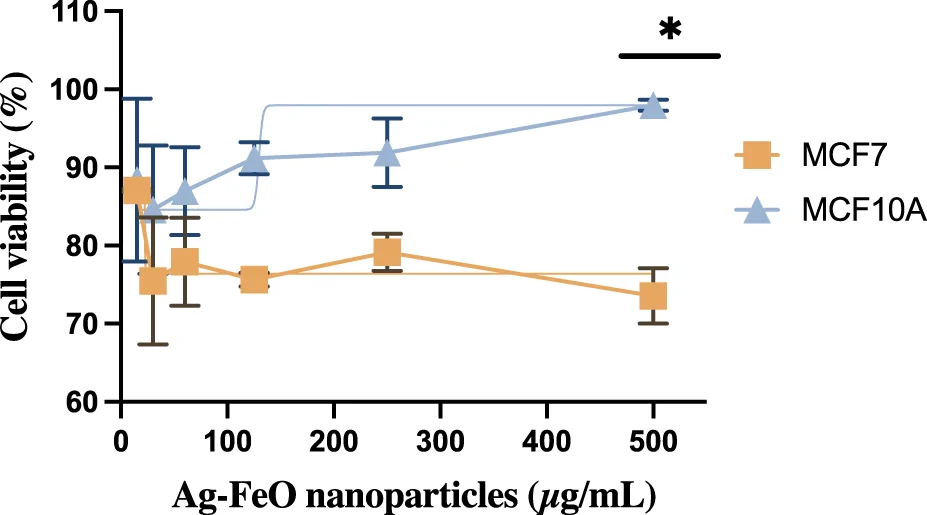
Effect of Ag-FeO NPs on the viability of MCF-7 breast cancer cells and MCF-10A non-tumorigenic mammary epithelial cells, as determined by the MTT assay. Cell viability is presented as mean ± SE following treatment with Ag-FeO NPs at concentrations ranging from 15 to 500 μg/mL. Solid lines represent nonlinear regression dose-response curves used for IC_50_ estimation. Statistical analysis was performed using two-way ANOVA followed by Šídák’s multiple comparisons test. A significant difference between MCF-7 and MCF-10A cells was observed only at 500 μg/mL (*P < 0.05).

### 3.8 In silico computational analysis

#### 3.8.1 Biological activity prediction using the PASS online platform

The following results represent computational predictions generated using PASS, SwissTargetPrediction, SwissADME, and ProTox-II platforms and should be interpreted as potential biological, pharmacokinetic, and toxicological properties rather than experimentally validated findings. The 2D chemical structures of the compounds identified from *Traganum nudatum* seed extract are illustrated in Figure 11. The PASS analysis results are presented in Table 2.

**FIGURE 11:**
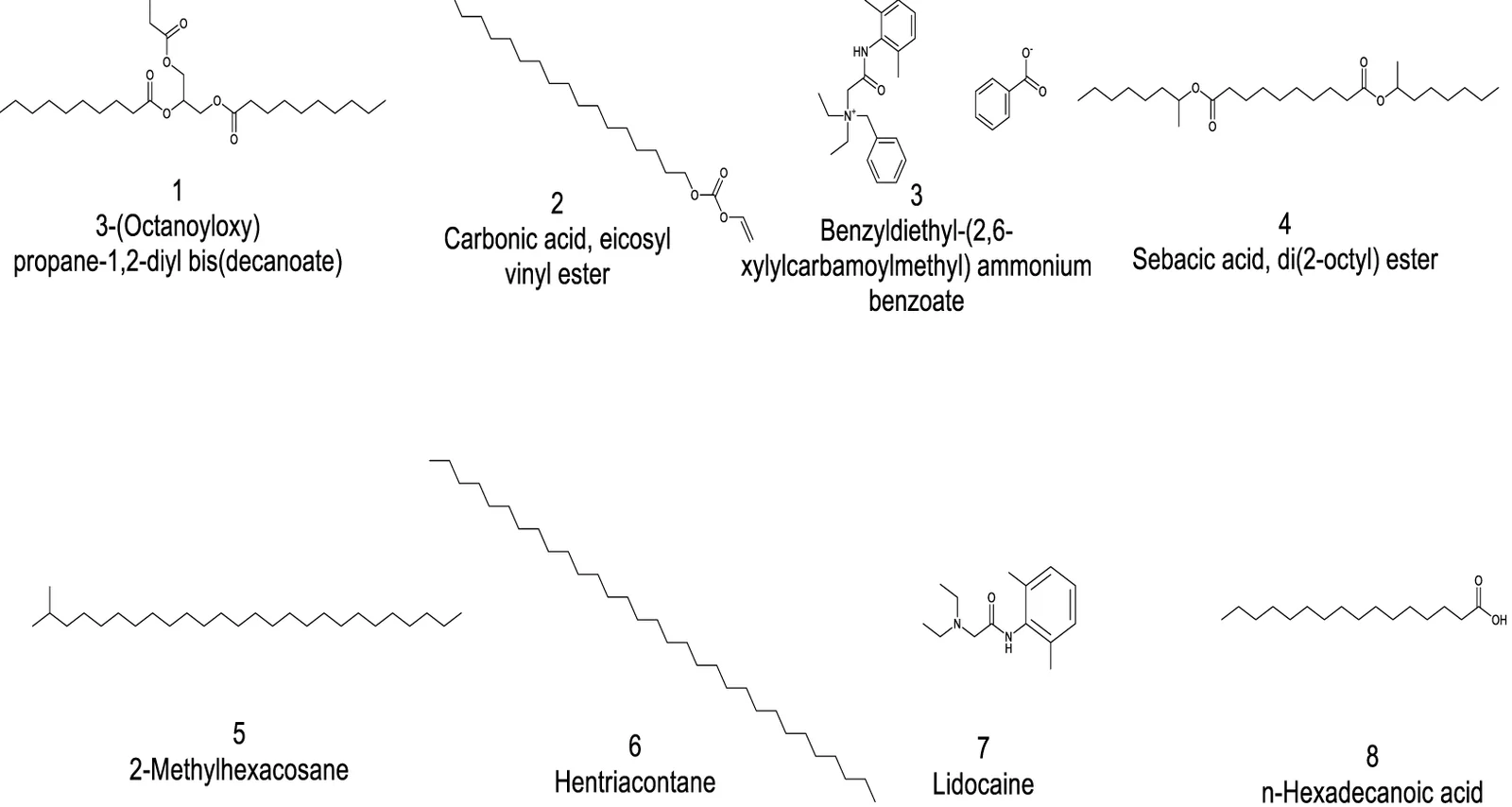
Chemical structures of the eight major compounds identified in *Traganum nudatum* seed extract by GC-MS analysis and selected for subsequent *in silico* evaluation using chemdraw Pro 21 software.

**TABLE 2:** Prediction of activity spectra for the eight major compounds identified in Traganum nudatum seed extract using PASS Online.

Compound number (name)	P_a_	P_i_	Predicted activity
1(3-(Octanoyloxy) propane-1,2-diyl bis(decanoate))	0.379	0.018	Antineoplastic antimetabolite
	0.247	0.085	Antibacterial
	0.113	0.033	Antifungal enhancer
2(Carbonic acid, eicosyl vinyl ester)	0.107	0.087	Antineoplastic antimetabolite
	0.418	0.026	Antibacterial
	0.505	0.030	Antifungal
3(benzyldiethyl-(2,6-xylylcarbamoylmethyl) ammonium benzoate)	NA	NA	NA
4(Sebacic acid, di(2-octyl) ester)	0.234	0.028	Antineoplastic antimetabolite
	0.293	0.063	Antibacterial
	0.129	0.015	Antifungal enhancer
5(2-Methylhexacosane)	0.179	0.037	Antineoplastic antimetabolite
	0.299	0.060	Antibacterial
	0.137	0.010	Antifungal enhancer
6(Hentriacontane)	0.234	0.028	Antineoplastic antimetabolite
	0.278	0.004	Antibacterial, ophthalmic
	0.124	0.019	Antifungal enhancer
7(Lidocaine)	0.184	0.116	Antineoplastic alkaloid
	0.152	0.053	Antibacterial, ophthalmic
	NA	NA	Antifungal
8(*n*-Hexadecanoic acid)	0.362	0.020	Antineoplastic antimetabolite
	0.300	0.060	Antibacterial
	NA	NA	Antifungal

NA: Not Available, activity prediction not provided in the PASS tool online.

PASS analysis indicated that compound 2 had the highest predicted probability of antifungal and antibacterial activity, with P_a_ values of 0.505 and 0.418, respectively, and low P_i_ values of 0.030 (antifungal) and 0.026 (antibacterial). These predictions suggest potential biological activity and support the prioritization of compound 2 for further experimental validation. Compounds one and eight showed moderate antineoplastic activity, with P_a_ values of 0.379 and 0.362, respectively. Additionally, compound 8 recorded a P_a_ value of 0.300 for antibacterial activity. All other tested compounds demonstrated low to moderate antibacterial potential, with P_a_ values below 0.5, indicating a relatively low probability of biological activity according to PASS prediction criteria (Filimonov et al., 2014). It is worth noting that, based on the PASS analysis, compound 3 showed no prediction (NA) when its SMILES string was entered on the PASS online website, indicating it lacks structural similarity to known bioactive compounds. Overall, compound 2 showed the highest predicted probabilities of antibacterial and antifungal activity among the analyzed compounds, while compound 1 exhibited the highest predicted probability of antineoplastic activity among the analyzed compounds (Figure 12).

**FIGURE 12:**
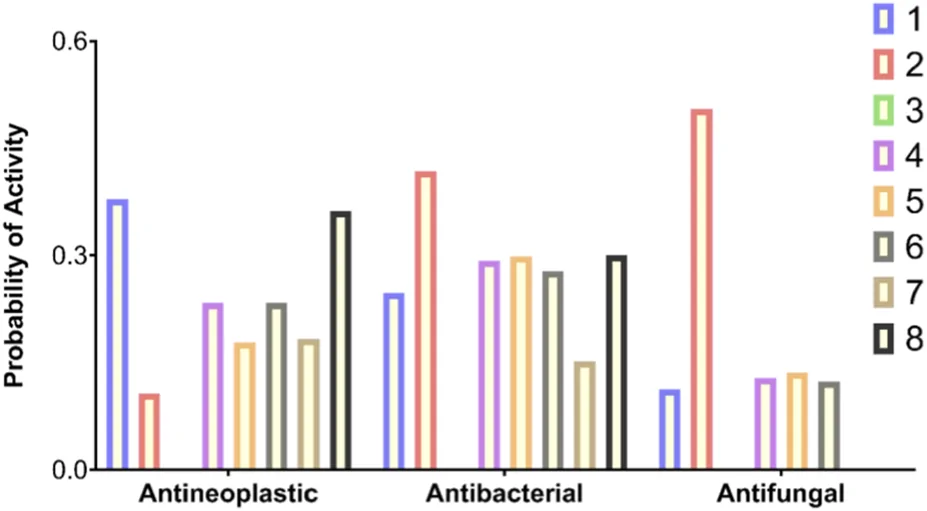
Predicted biological activities of the eight major compounds identified in *Traganum nudatum* seed extract using the PASS online platform.

#### 3.8.2 Molecular target prediction using the SwissTargetPrediction website

The molecular target prediction results obtained using the SwissTargetPrediction platform are presented in Figure 13 and Table 3. For compound 1, kinases represented the most prominent target class (40.0%), suggesting potential interactions with kinase-related proteins. Other enzymes accounted for 33.3% of the predicted targets, indicating possible interactions with additional enzyme classes. Overall, approximately 73.3% of the predicted targets were enzyme-related, while the remaining classes each represented 6.7% of the total predicted targets.

**FIGURE 13:**
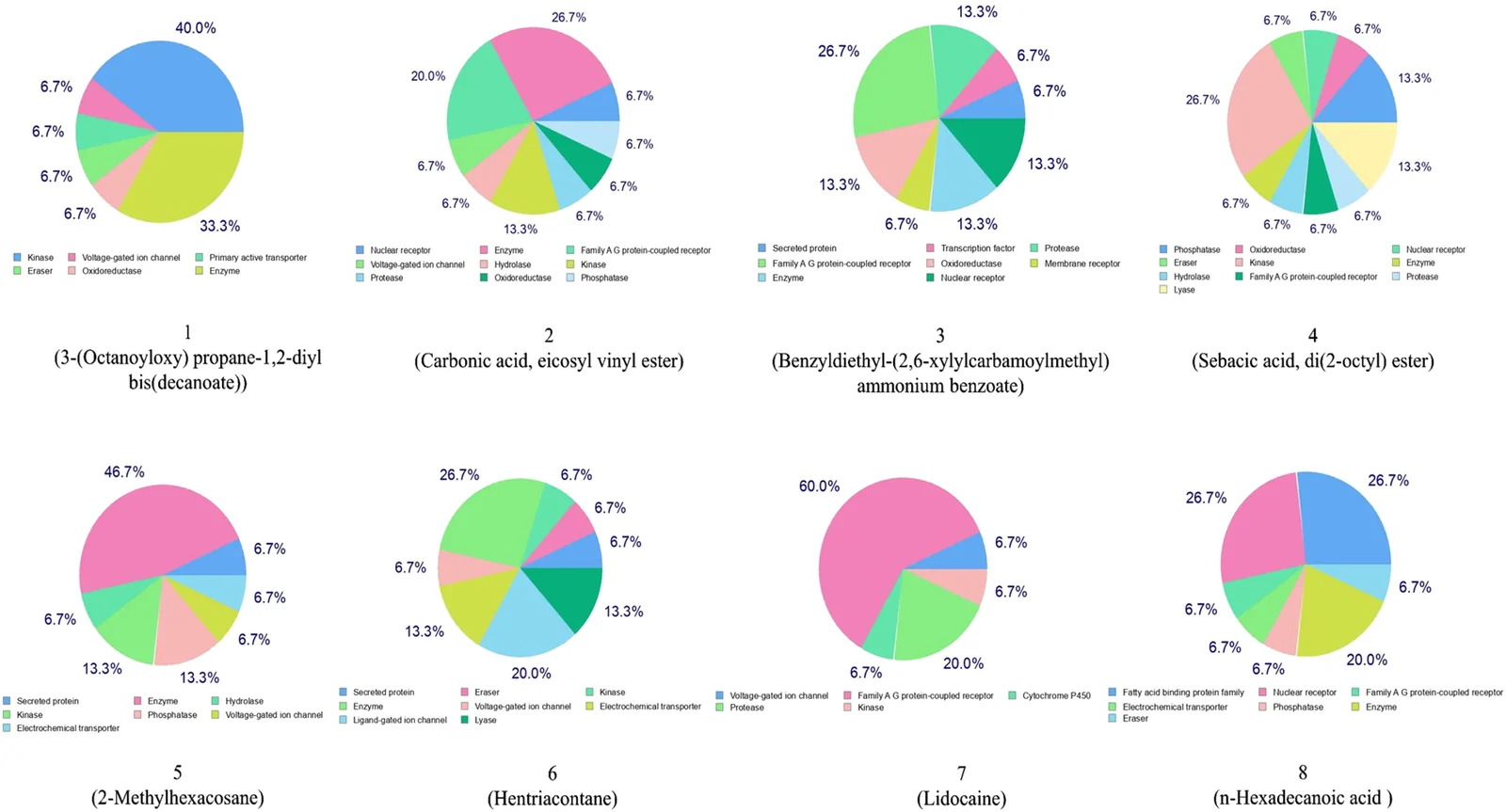
Distribution of predicted target classes for the eight major compounds identified in *Traganum nudatum* seed extract using SwissTargetPrediction.

**TABLE 3:** Predicted molecular targets of the eight major compounds identified in *Traganum nudatum* seed extract using SwissTargetPrediction.

Compound number	Target	Target class	Probability
1	Protein kinase C isoforms (alpha, epsilon, theta, and delta), and (eta, gamma by homology)	Kinase	0.37
2	No targets predicted	-	-
3	All predicted targets showed equal probability from various classes	​	0.10
4	Dual specificity phosphatase Cdc25A	Phosphatase	0.15
5	Testis-specific androgen-binding protein	Secreted protein	0.13
6	Testis-specific androgen-binding protein	Secreted protein	0.13
7	Sodium channel protein type IV alpha subunit	Voltage-gated ion channel	1.00
8	Fatty acid binding proteins (adipocyte, muscle, epidermal, and intestinal)	Fatty acid binding protein family	0.94
	Peroxisome proliferator-activated receptor (alpha, and delta)	Nuclear receptor	

No molecular targets were predicted for compound 2. For compound 3, the dominant target class was Family A G protein-coupled receptors (26.7%). Proteases, oxidoreductases, enzymes, and nuclear receptors each accounted for 13.3%, whereas secreted proteins, transcription factors, and membrane receptors were represented at 6.7%, suggesting a broader range of potential molecular interactions.

Compound 4 was predicted to interact primarily with kinases (26.7%), followed by phosphatases and lyases (13.3% each). The remaining target classes were distributed among several protein families at lower percentages. For compound 5, enzymes constituted the largest target class (46.7%), followed by kinases and phosphatases (13.3% each), while the remaining target classes each accounted for 6.7%.

For compound 6, enzymes were again the predominant target class (26.7%), followed by ligand-gated ion channels (20.0%) and lyases (13.3%). Compound 7 was predicted to preferentially interact with Family A G protein-coupled receptors, which represented 60.0% of its predicted targets, while proteases accounted for 20.0%. Finally, compound 8 showed equal representation of fatty acid-binding proteins and nuclear receptors (26.7% each), followed by enzymes (20.0%). The remaining target classes were each represented at 6.7%.

Overall, the predicted targets were distributed across multiple protein classes, suggesting that the analyzed compounds may interact with diverse biological pathways and molecular processes.

#### 3.8.3 ADME property prediction using the SwissADME website

The pharmacokinetic properties of eight selected compounds were evaluated using the SwissADME website, which focuses on several important parameters, including Molecular Weight, lipophilicity (Log *P*_*o/w*_), solubility (Log *S*), blood-brain barrier (BBB) penetration, gastrointestinal (GI)/oral absorption, and violation of Lipinski’s rule of five (ROF). The results showed variation among the study compounds, as shown in Table 4 and Figure 14. The molecular weights of all compounds were within the suitable range (<500 g/mol) according to Lipinski’s rules, except for compound 1, which exceeded the limit. Compounds 3, 7, and eight were predicted to exhibit relatively favorable pharmacokinetic characteristics, including high gastrointestinal absorption and BBB permeability. Compounds 3 and 7 showed no Lipinski’s rule violations, whereas compound 8 showed one violation. Their lipophilicity (Log Po/w) and solubility (Log S) values were also relatively favorable.

**TABLE 4:** Predicted ADME properties of the eight major compounds identified in Traganum nudatum seed extract using SwissADME.

Compound number	Molecular weight	Log *Po/w*	Log *S*	GI absorption	BBB permeant	Lipinski
1	526.79 g/mol	8.63	−9.66	Low	No	No; 2 violations: MW > 500, MLOGP>4.15
2	368.59 g/mol	8.32	−7.78	Low	No	Yes; 1 violation: MLOGP>4.15
3	446.58 g/mol	4.01	−8.11	High	Yes	Yes; 0 violation
4	426.67 g/mol	7.91	−8.09	Low	No	Yes; 1 violation: MLOGP>4.15
5	380.73 g/mol	10.63	−10.34	Low	No	Yes; 1 violation: MLOGP>4.15
6	436.84 g/mol	12.34	−12.28	Low	No	Yes; 1 violation: MLOGP>4.15
7	234.34 g/mol	2.39	−4.5	High	Yes	Yes; 0 violation
8	256.42 g/mol	5.55	−5.31	High	Yes	Yes; 1 violation: MLOGP>4.15

**FIGURE 14:**
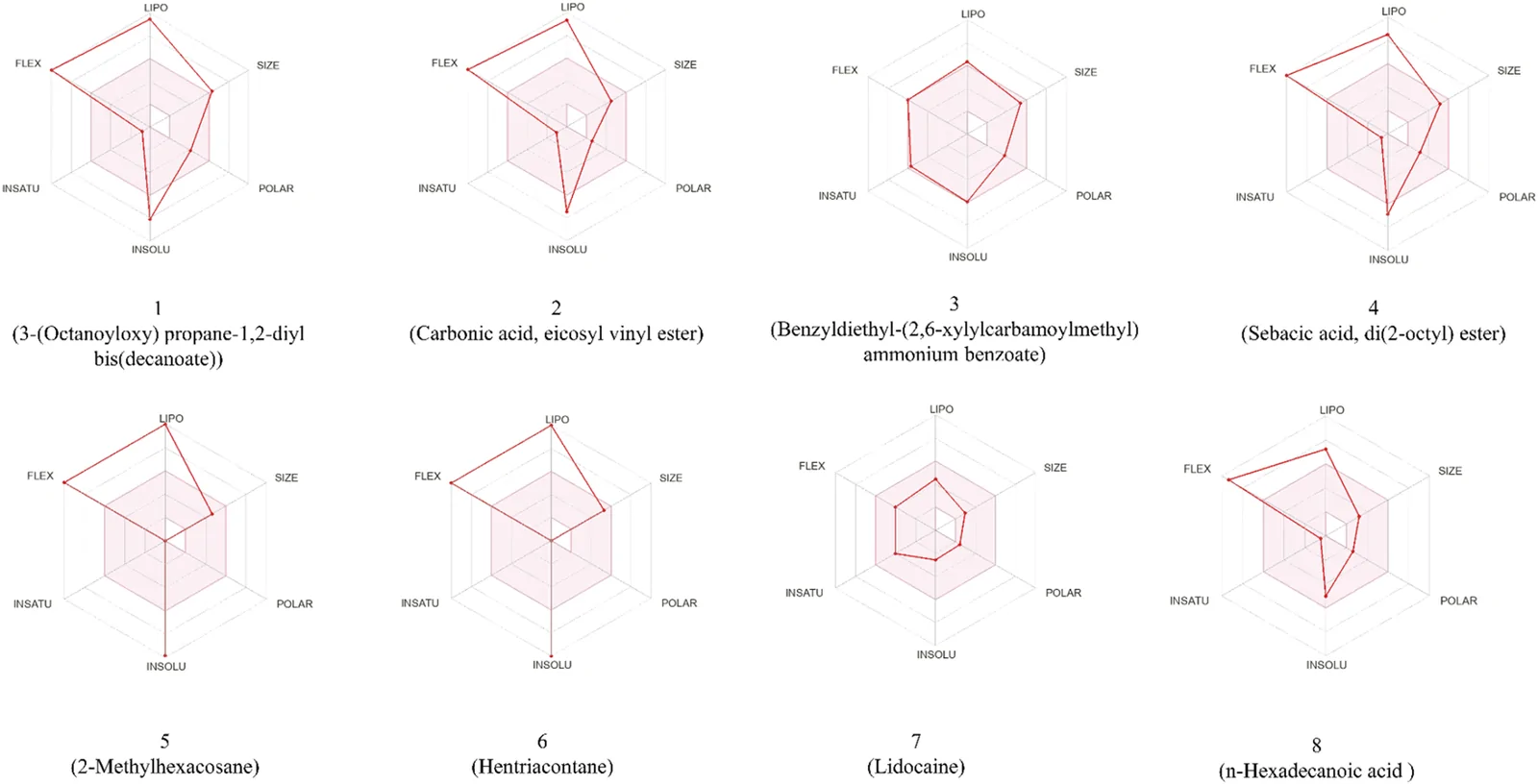
Radar charts for oral bioavailability of the identified compounds in *Traganum nudatum* seed extract using the SwissADME website. Abbreviations: “LIPO, lipophilicity; SIZE, molecular size; POLAR, polarity; INSOLU, insolubility; INSATU, insaturation; FLEX, flexibility”.

In contrast, compounds 1, 5, and 6 exhibited less favorable pharmacokinetic properties, including high lipophilicity (Log _*Po/*_w), very low solubility (Log S), poor gastrointestinal absorption, lack of BBB permeability, and Lipinski’s rule violations.

Compounds 2 and 4 exhibited only one Lipinski’s rule violation, low gastrointestinal absorption, and high lipophilicity (Log *P*_*o/w*_). These predictions indicate that compounds 3, 7, and 8 may be suitable candidates for further pharmacological evaluation and can be considered potential candidates for further biological and experimental studies, whereas the other compounds may need structural modifications to improve their properties. The SwissADME bioavailability radar plots (Figure 14) revealed notable differences among the analyzed compounds. Compounds 3 and 7 exhibited the most favorable drug-like profiles, with physicochemical properties largely within the optimal bioavailability range. Compound 8 showed minor deviations, whereas compounds 1, 5, and 6 displayed less favorable characteristics, mainly related to high lipophilicity and poor solubility. Compounds 2 and 4 demonstrated intermediate properties. Overall, the radar analysis was consistent with the pharmacokinetic predictions in Table 4 and identified compounds 3 and 7 as the most promising candidates for further investigation.

#### 3.8.4 Prediction of cytochromes P450 (CYP) inhibition profile using the SwissADME website

Prediction of potential interactions between the studied compounds and CYP450 enzymes, which play a major role in drug metabolism, revealed differences in their predicted inhibition profiles (Table 5). Compounds 2, 4, and eight were predicted to inhibit CYP1A2, whereas none of the compounds was predicted to inhibit CYP2C19. Compounds 4 and eight showed predicted inhibition of CYP2C9, while compounds 3 and 7 were predicted to inhibit CYP2D6. In addition, compounds one and 4 were predicted to inhibit CYP3A4. Overall, the compounds exhibited distinct CYP450 inhibition patterns, suggesting differences in their potential metabolic interactions. Among the tested compounds, compound 4 was predicted to inhibit CYP1A2, CYP2C9, and CYP3A4, whereas compounds 5 and 6 were not predicted to inhibit any of the CYP450 isoforms evaluated in this study. However, these predictions are limited to the selected CYP450 enzymes and should not be interpreted as a comprehensive assessment of metabolic activity or drug-drug interaction potential.

**TABLE 5:** Predicted cytochrome P450 inhibition profile of *Traganum nudatum* seed extract using SwissADME.

CYP450 enzymes	Compound number							
	1	2	3	4	5	6	7	8
CYP1A2	No	Yes	No	Yes	No	No	No	Yes
CYP2C19	No	No	No	No	No	No	No	No
CYP2C9	No	No	No	Yes	No	No	No	Yes
CYP2D6	No	No	Yes	No	No	No	Yes	No
CYP3A4	Yes	No	No	Yes	No	No	No	No

#### 3.8.5 Toxicity prediction using the ProTox-II website

The toxicity profiles of eight selected compounds were evaluated using the ProTox-II platform. The analysis included predictions of hepatotoxicity, carcinogenicity, immunotoxicity, mutagenicity, and cytotoxicity. As shown in Table 6, all compounds were predicted to be inactive for hepatotoxicity, with probabilities ranging from 62% to 97%, indicating a low potential risk of liver toxicity. Compounds 3, 5, 6, 7, and eight were predicted to be inactive across all evaluated toxicity endpoints and exhibited favorable safety profiles. In contrast, compound 1 was predicted to exhibit carcinogenicity (74%) and mutagenicity (68%), suggesting potential safety concerns that require further experimental validation. Similarly, compounds 2 and 4 were predicted to possess carcinogenic potential, with probabilities of 57% and 60%, respectively. Overall, the ProTox-II predictions suggest that compounds 3, 5, 6, 7, and eight have lower predicted toxicity risks compared with the other compounds. In contrast, compounds 1, 2, and 4 showed predicted activity for one or more toxicity endpoints, particularly carcinogenicity, suggesting that these compounds warrant further experimental safety evaluation.

**TABLE 6:** Predicted toxicity profiles of selected compounds identified in *Traganum nudatum* seed extract using ProTox-II.

Compound number	​				
​	Hepatotoxicity (%)	Carcinogenicity (%)	Immunotoxicity (%)	Mutagenicity (%)	Cytotoxicity (%)
1	Inactive (88)	Active (74)	Inactive (98)	Active (68)	Inactive (86)
2	Inactive (78)	Active (57)	Inactive (98)	Inactive (98)	Inactive (79)
3	Inactive (87)	Inactive (64)	Inactive (99)	Inactive (70)	Inactive (60)
4	Inactive (62)	Active (60)	Inactive (95)	Inactive (97)	Inactive (79)
5	Inactive (77)	Inactive (72)	Inactive (98)	Inactive (94)	Inactive (77)
6	Inactive (74)	Inactive (58)	Inactive (98)	Inactive (100)	Inactive (78)
7	Inactive (97)	Inactive (71)	Inactive (98)	Inactive (73)	Inactive (59)
8	Inactive (52)	Inactive (63)	Inactive (99)	Inactive (100)	Inactive (74)

The percentages represent the predicted probability values provided by the ProTox-II platform.

## 4 Discussion

In this study, Ag-FeO NPs were successfully synthesized using the seed extract of *T. nudatum*. Conventional nanoparticle synthesis methods typically rely on physical and chemical approaches that may pose risks to the environment and biological systems due to the use of toxic reagents and harsh conditions (Osman et al., 2024). In contrast, biological synthesis offers an economical, sustainable, and environmentally benign alternative, making it a highly promising strategy for nanoparticle production (Suppiah et al., 2023). Plant extracts are particularly attractive for green nanoparticle synthesis because of their rich phytochemical profiles, which act as natural reducing and stabilizing agents (Singh et al., 2023). Consistent with previous reports, plant extracts have been successfully employed for nanoparticle synthesis from diverse botanical sources, including *Adansonia digitata* fruit shells (Aldahasi et al., 2024), *Vernonia amygdalina* (Tesfaye et al., 2023), and Acer oblongifolium (Naveed et al., 2022), supporting the feasibility and versatility of plant-mediated nanoparticle synthesis.

The synthesized bimetallic Ag-FeO NPs were initially characterized using UV-Vis spectroscopy to assess their optical properties and provide evidence of nanoparticle formation. A characteristic absorption peak was observed at 359 nm, supporting the formation of Ag-FeO NPs.

This finding is consistent with previous reports, including Devadoss et al. (2023), who observed a comparable absorption band for bimetallic nanoparticles. Similarly, the biosynthesis of bimetallic nanoparticles using *Lagenaria siceraria* leaf extract exhibited an absorption peak at approximately 330 nm (Elkady et al., 2025). In contrast, bimetallic nanoparticles synthesized using *Artemisia abyssinica* leaf extract showed a slightly red-shifted absorbance peak at 380 nm (Orshiso et al., 2023). Variations in absorption peaks among studies may be attributed to differences in the plant materials used, phytochemical composition, particle size, and metal ratios.

Comparative UV-Vis analysis of plant extracts, precursor solutions, and synthesized nanoparticles can strengthen the interpretation of spectral changes associated with nanoparticle formation (Kanthireegala et al., 2025). Although such comparative spectra were not obtained in the present study, the absorption feature observed for the Ag-FeO NPs, together with complementary physicochemical characterization, supports their formation. Nevertheless, the absence of these comparative spectra represents a limitation and precludes definitive interpretation of the observed spectral feature based on UV-Vis analysis alone.

The surface morphology of the synthesized Ag-FeO NPs was examined after optimizing the synthesis conditions using *Traganum nudatum* seed extract. Scanning electron microscopy (SEM) images revealed that the nanoparticles were relatively uniformly dispersed, although their individual shapes were not clearly defined. A similar uniformly dispersed morphology has been reported for green-synthesized iron nanoparticles using green tea extract (Hao et al., 2021).

Although the EDX spectrum qualitatively indicated the presence of the principal elements Ag, Fe, and O, quantitative elemental data, including weight percentage (wt%) and atomic percentage (at%), were not available. Consequently, the relative elemental proportions and material purity could not be determined, representing a limitation of the present study. Therefore, the EDX findings should be interpreted as qualitative evidence of elemental presence rather than quantitative confirmation of nanoparticle composition and purity.

In contrast, transmission electron microscopy (TEM) analysis showed that the Ag-FeO NPs predominantly exhibited rod-like morphologies, while some particles appeared more spherical. Notably, a degree of particle agglomeration was observed, which hindered precise shape identification. Comparable morphological diversity has been reported in the green synthesis of Au-Ag-core-shell nanoparticles using *Garcinia mangostana* peel extract, where predominantly spherical nanoparticles with occasional irregular and rod-shaped forms were observed (Lee et al., 2022).

Additional characterization of Ag-FeO NPs formation using *Traganum nudatum* seed extract was obtained through dynamic light scattering (DLS) and zeta potential analysis. The magnitude of the zeta potential is an important indicator of nanoparticle surface charge and colloidal stability, with values between −30 and +30 mV generally considered indicative of electrostatic stabilization (Agmo Hernández, 2023). In the present study, the Ag-FeO NPs exhibited a zeta potential of −1.36 mV. Although relatively low in magnitude, the negative surface charge suggests interparticle repulsion that contributes to colloidal stability, consistent with previous findings (Kordy et al., 2022). Comparable results have been reported for bimetallic nanoparticles synthesized using *Ocimum basilicum* leaf extract, where AgPt nanoparticles exhibited a zeta potential of −16 mV.

The low zeta potential value of −1.36 mV indicates weak electrostatic repulsion between Ag-FeO NPs, which may reduce colloidal stability and promote partial particle aggregation. This observation is consistent with the aggregation tendency observed in the TEM and SEM micrographs. The relatively high PDI value (0.60) further supports the presence of a broad particle size distribution. Such aggregation may be attributed to weak surface charge, plant-derived capping molecules, and possible magnetic interactions associated with the iron oxide component (Radulescu et al., 2023). Therefore, although Ag-FeO NPs were successfully synthesized, their colloidal stability appears limited under the tested conditions.

Fourier-transform infrared (FTIR) analysis was employed to characterize the surface chemistry of the synthesized Ag-FeO NPs and to confirm the presence of phytochemical residues associated with the *Traganum nudatum* seeds extract used in the green synthesis process. The FTIR spectra of the phyto-synthesized Ag-FeO NPs exhibited prominent absorption bands at 3,315 and 1,645 cm^-1^. The broad band observed around 3,315 cm^-1^ corresponds to O-H stretching vibrations of hydroxyl groups from alcohols and phenolic compounds, while the peak at 1,645 cm^-1^ is attributed to C=O stretching vibrations of carboxylic groups and N-H bending vibrations of amine groups present in biomolecules from the seed extract (Ghosh and Singh, 2021). These functional groups are indicative of plant-derived phytochemicals that remain attached to the nanoparticle surface following synthesis (Ansari et al., 2021). Moreover, their presence supports the role of these biomolecules as capping and stabilizing agents during the green synthesis process, where they help prevent particle aggregation through steric and electrostatic stabilization, thereby contributing to improved colloidal stability of the nanoparticles (Berta et al., 2021).

During green synthesis, some phytochemicals are consumed in the reduction of metal ions, whereas others remain adsorbed on the nanoparticle surface as capping and stabilizing agents (Singh et al., 2023). However, because post-synthesis GC-MS analysis was not performed, the presence of individual free phytochemicals in the final nanoparticle preparation could not be confirmed. Therefore, the proposed role of these compounds is based on the FTIR evidence and previous reports on plant-mediated nanoparticle synthesis (Ansari et al., 2021).

The phytochemical profile identified in this study aligns with previous research on *Traganum nudatum*, which has revealed a diverse array of secondary metabolites such as terpenoids, fatty acid methyl esters, sterols, alkaloids, and phenolic compounds. Key constituents reported include carvacrol, citronellol, elaidic acid, stearic acid, palmitic acid, oleic acid, and linoleic acid. Additionally, analyses have shown abundant saturated and unsaturated fatty acids, particularly behenic and palmitic acids, along with various phenolic metabolites. These results confirm *T. nudatum* as a rich source of biologically active compounds, supporting its known antioxidant, anti-inflammatory, antidiabetic, and other pharmacological effects (Benhassine et al., 2021). While the overall phytochemical composition appears stable, variations may arise due to factors such as geographical origin, environmental conditions, plant developmental stage, extraction techniques, and analytical methods (El-bassossy, 2022).

Furthermore, the synthesized Ag-FeO NPs demonstrated antimicrobial activity against the tested pathogens in the order *Escherichia coli* < methicillin-resistant *Staphylococcus aureus* (MRSA) < *Candida albicans*. These findings are consistent with those of Hassan et al. (2025), who reported significant antimicrobial activity of Ag-Fe bimetallic nanoparticles synthesized using *Khaya senegalensis* leaf extract, particularly against *E. coli* and *Candida albicans* at concentrations ranging from 10 to 30 mg/mL. Similarly, Yahaya et al. (2023) demonstrated that bimetallic Ag-Cu nanoparticles synthesized from *Celtis integrifolia* leaf extract exhibited enhanced antimicrobial activity compared with plant extracts alone at concentrations between 100 and 500 μg/mL. Overall, bimetallic nanoparticles have consistently exhibited significant antimicrobial performance across different studies and microbial species, suggesting their potential as effective antimicrobial agents due to their enhanced physicochemical characteristics and multifaceted mechanisms of action (Arora et al., 2020).

Although the antimicrobial activity of Ag-FeO NPs was lower than that of the positive controls, the nanoparticles exhibited measurable inhibitory effects against both bacterial and fungal pathogens. These findings suggest that Ag-FeO NPs may serve as an alternative antimicrobial agent, warranting further optimization and mechanistic investigation. Further, the use of ampicillin as the positive control for methicillin-resistant *Staphylococcus aureus* (MRSA) represents a limitation of the present study since MRSA is intrinsically resistant to β-lactam antibiotics (Chambers and DeLeo, 2009).

The SEM findings presented in the results are closely aligned with stress response mechanisms described for *Candida albicans*. The observed transition from smooth, intact cells in the control group to rough, deformed, and pitted cell surfaces in the treated samples indicates severe disruption of cell wall integrity induced by Ag-FeO NPs. The presence of aggregated cells and filamentous structures resembling hyphae or pseudohyphae further suggests activation of stress-induced morphological adaptation (Fekete et al., 2007). This is consistent with the known ability of *C. albicans* to alter its growth form under environmental stress conditions as a survival strategy (Rahmawati Setiadi aus Leverkusen et al., 2006). This response reflects an attempt to adapt to nanoparticle-induced damage, particularly oxidative and structural stress affecting the cell envelope. In contrast, such pronounced morphological transitions are not typically expected in bacterial species such as *Escherichia coli* and MRSA. While both organisms activate stress-response pathways under nanoparticle exposure, their structural organization differs significantly from fungi. *Escherichia coli*, as a Gram-negative bacterium, primarily responds through outer membrane remodeling and efflux mechanisms, whereas MRSA relies on cell wall thickening and enzymatic defense systems. Therefore, unlike *C. albicans*, these bacteria are less likely to exhibit filamentation or pseudohyphal-like morphological switching, although membrane disruption and surface damage may still occur under similar stress conditions (Dumeaux et al., 2023).

The MTT assay results demonstrated that Ag-FeO NPs exerted greater cytotoxic effects on MCF-7 breast cancer cells than on MCF-10A normal breast epithelial cells. MCF-7 cells exhibited lower viability values (73.56%–87.06%) than MCF-10A cells (84.59%–97.96%), indicating increased susceptibility of cancer cells to nanoparticle exposure. Two-way ANOVA revealed a significant effect of cell type on viability (P = 0.0022), whereas neither concentration nor the interaction between concentration and cell type significantly affected cell viability, indicating the absence of a clear concentration-dependent response within the tested range. Pairwise comparisons showed a significant difference between the 2 cell lines only at 500 μg/mL (P = 0.0447). Nevertheless, the consistently higher viability of MCF-10A cells suggests differential sensitivity between the 2 cell lines, with MCF-7 cells showing greater susceptibility to Ag-FeO NP exposure under the tested conditions.

A similar pattern has been reported by Hashem et al. (2023), who described selective anticancer activity of biosynthesized selenium-silver (Se-Ag) bimetallic nanoparticles against MCF-7 cells, with comparatively lower toxicity toward normal cells.

Recent advances in nanomedicine have highlighted engineered nanoparticles as versatile platforms for cancer treatment because they can improve tumor targeting, enhance therapeutic delivery, minimize off-target toxicity, and regulate molecular pathways involved in tumor progression and angiogenesis. These properties enable nanoparticle-based systems to address several limitations of conventional cancer therapies while improving therapeutic outcomes (Kanthireegala et al., 2026; Lionadi and Payam, 2026).

In this context, the differential cytotoxic response observed between MCF-7 and MCF-10A cells suggests that the biosynthesized Ag-FeO NPs warrant further investigation for potential biomedical applications; however, additional dose-response studies and appropriate extract controls are required before conclusions regarding selective anticancer activity can be established. The observed biological activities of the Ag-FeO NPs may reflect contributions from both the nanoparticle components and plant-derived phytochemicals associated with their surface, as suggested by the FTIR analysis. However, because the *T. nudatum* seed extract was not evaluated independently in the MTT assay, the relative contribution of the seed-derived constituents to the observed cytotoxic effects cannot be distinguished from that of the Ag-FeO NP formulation. Therefore, the present findings do not establish whether nanoparticle synthesis enhanced the cytotoxic activity relative to the seed extract alone. Future studies should directly compare the seed extract and Ag-FeO NPs under identical experimental conditions to clarify their respective contributions to the observed cytotoxic response.

FTIR analysis of Ag-FeO NPs and GC-MS analysis of seed extract revealed plant-derived functional groups and metabolites, respectively, suggesting that phytochemicals from *T. nudatum* remained associated with the nanoparticle surface and contributed to reduction and stabilization. However, because post-synthesis GC-MS analysis was not performed, the presence and abundance of individual metabolites in the final nanoparticle formulation could not be confirmed. Thus, the *in silico* results should be considered supportive rather than definitive evidence of their contribution to the observed biological activities.

The results of the PASS analysis indicated that compound 2 has the highest potential for antibacterial and antifungal activities, making it a promising candidate for the development of natural antimicrobials. These results were consistent with a study that reported, through GC-MS analysis, the secretion of antifungal metabolites by two endophytes, including carbonic acid, eicosyl vinyl ester (Chowhan and Chakraborty, 2024). In addition, carbonic acid, eicosyl vinyl ester, is a compound detected through GC-MS analysis in the methanolic extract of *Cynometra ramiflora* that potentially contributes to its antioxidant properties (Samadd et al., 2024).

Compounds one and eight showed moderate predicted antineoplastic activity. This finding is consistent with previous reports showing that n-hexadecanoic acid can induce apoptosis in triple-negative breast cancer cells (Sangpairoj et al., 2022), and exhibits antioxidant and antibacterial activities (Ganesan et al., 2024).

Compound 1 (3-(octanoyloxy) propane-1,2-diyl bis(decanoate)) was identified in coconut oil by GC-MS and has been reported to exhibit antiviral, antibacterial, and antifungal activities (Elnosary et al., 2024). Overall, these results highlight compound 2 as the most promising candidate for antibacterial and antifungal activities and compound 1 as the compound with the highest predicted probability of antineoplastic activity. However, studies investigating its anticancer activity remain limited. Further biological studies are needed to confirm these predictions and move to the experimental evaluation stage. Compound 7 (lidocaine) demonstrated a dose-dependent antibacterial effect (Heller et al., 2025) providing significant inhibition of *Staphylococcus aureus* and MRSA after 12 h at 2% concentration. A reduction in tumor proliferation was observed in triple-negative breast cancer with lidocaine, as well as decreased Bcl-2 activity and increased p53, which supports the induction of apoptosis (Long et al., 2022).

The results of the molecular target prediction of the eight compounds using the SwissTargetPrediction website revealed a variability in the target protein classes, with some important specific targets that contributed to understanding the potential biochemical mechanisms of these compounds. SwissTargetPrediction was employed to predict the potential protein targets of the major phytochemicals identified by GC-MS. By identifying multiple protein targets and target classes, this approach provides broader mechanistic insight into the observed antimicrobial and anticancer activities than analyses focused on a single molecular target. However, these predicted targets were not validated by molecular docking. Future studies should therefore perform molecular docking using selected protein targets for providing additional mechanistic support for the observed biological activities.

The kinase class was one of the potential targets, specifically protein kinase C (alpha, epsilon, theta, delta, eta, and gamma) enzymes, that play a main role in cell proliferation. This is consistent with studies that have indicated the role of PKC inhibitors as anticancer agents and modulators of signaling pathways (Liu et al., 2012; Yin et al., 2019), suggesting that compounds targeting this class may be effective as inhibitors of PKC-dependent signaling pathways specifically in cancer cells. The results also showed that G protein-coupled receptors (GPCRs) were potential targets, suggesting their potential impact on cancer cell growth pathways. This is consistent with the study that demonstrated that GPCRs were targets of flavonoids in cancer therapy by regulating pathways such as MAPK and PI3K/Akt and inducing apoptosis (Sak, 2024). The compounds also targeted the phosphatase class, specifically Cdc25A, a phosphatase enzyme involved in cell cycle regulation. Overexpression of Cdc25A has been associated with the development of various cancers, making it an important therapeutic target (Dakilah et al., 2024).

Regarding the SwissADME analysis of the eight compounds, they demonstrated a clear variation in pharmacokinetic potential. The tool’s prediction indicated that compounds 3, 7, and eight met Lipinski’s criteria and exhibited good gastrointestinal absorption, the ability to cross the blood-brain barrier (BBB), and acceptable solubility values, making them promising candidates for further experimental investigation. Compounds that adhere to Lipinski’s criteria are ideal drug candidates and more likely to succeed in early drug development (Nhlapho et al., 2024). SwissADME represents a powerful tool in the early phase of drug design that helps to select compounds with the best ADME potential for further biological studies to improve their properties (Dulsat et al., 2023). Regarding CYP450, SwissADME predicted that compound 4 possesses potential for inhibiting CYP450 enzymes that require special attention for potential drug interactions during clinical development. On the other hand, compounds 5 and 6 showed no inhibitory effect on CYP450 enzymes, making them safer candidates for drug development. It is well known that inhibition of CYP450 enzymes can lead to drug-drug interactions (DDIs) due to drug accumulation or reduced metabolic clearance, thereby increasing the risk of toxicity and therapeutic failure (Gilani and Cassagnol, 2021; Lee et al., 2024; Rudik et al., 2022).

ProTox-II predicted all eight compounds to be inactive for hepatotoxicity, suggesting favorable preliminary hepatotoxicity profiles, indicating good preliminary safety profiles in terms of hepatotoxicity. The results also showed that compounds 3, 5, 6, 7, and eight were predicted to be inactive for carcinogenicity, immunotoxicity, mutagenicity, and cytotoxicity, indicating a high safety profile and supporting their potential for drug development. This evaluation highlights the effectiveness of the ProTox-II platform in distinguishing compounds with low predicted toxicity from those with high-risk potential. This capability is achieved through model training on both *in vitro* and *in vivo* data, which enhances the reliability of these predictions (Banerjee et al., 2024).

Overall, the *in silico* analyses support the experimentally observed antimicrobial and anticancer activities of the *Traganum nudatum*-mediated Ag-FeO NPs. The predicted protein targets, together with the favorable pharmacokinetic properties and low predicted toxicity of several major phytochemicals, provide potential mechanistic insight into how these compounds may contribute to the biological activity of the biosynthesized nanoparticles. Although the observed biological effects are likely the result of the combined action of the nanoparticles and their associated phytochemicals, the computational predictions should be regarded as supportive rather than definitive evidence of the contribution of individual phytochemicals.

## 5 Study limitations

The present study has several limitations. GC-MS analysis was performed only on the *Traganum nudatum* seed extract prior to nanoparticle synthesis; therefore, the phytochemical composition of the final Ag-FeO NPs was not determined. In addition, the seed extract was not evaluated independently, preventing direct assessment of its contribution to the observed biological activities.

The absence of comparative UV-Vis spectra of the seed extract and metal precursors represents a limitation; future studies should include these controls to strengthen the interpretation of nanoparticle formation. Quantitative EDX data (weight % and atomic %) were also unavailable, limiting comparison with previously reported green-synthesized Ag-FeO NPs. Antimicrobial activity was assessed only by the agar well diffusion method, as MIC, MBC, and MFC assays were not performed. Furthermore, the use of ampicillin as the positive control for MRSA should be interpreted with caution because MRSA is intrinsically resistant to β-lactam antibiotics. Finally, the anticancer activity was evaluated over a limited concentration range, and the relatively low *R* ^2^ values indicated a poor fit of the nonlinear regression model used to estimate IC_50_ values. Future studies should include post-synthesis phytochemical characterization, quantitative EDX analysis, independent evaluation of the seed extract, comprehensive antimicrobial susceptibility testing, appropriate anti-MRSA controls, mechanistic investigations, and *in vivo* validation. Future studies should incorporate molecular docking using biologically relevant target proteins to validate the predicted targets and characterize ligand-protein interactions underlying the observed biological activities.

## 6 Conclusion

The present study successfully synthesized bimetallic Ag-FeO NPs using *T. nudatum* seed extract, providing an environmentally friendly alternative to conventional physical and chemical synthesis methods. The synthesized Ag-FeO NPs were characterized using UV-Vis spectroscopy, FTIR, SEM, TEM, DLS, zeta potential analysis, and qualitative EDX analysis, collectively supporting nanoparticle formation and the involvement of plant-derived biomolecules in stabilization. However, because quantitative EDX data were unavailable, the relative elemental composition and purity of the synthesized material could not be established by EDX. Ag-FeO NPs exhibited notable antimicrobial activity against methicillin-resistant *Staphylococcus aureus* (MRSA), *Candida albicans*, and *Escherichia coli*. In the MTT assay, Ag-FeO NPs produced greater reductions in MCF-7 breast cancer cell viability than in MCF-10A non-tumorigenic breast epithelial cells under the tested conditions; however, a clear concentration-dependent response was not observed. Because the *T. nudatum* seed extract was not evaluated independently in the MTT assay, the relative contributions of the nanoparticle formulation and seed-derived constituents to the observed cytotoxic effects cannot be distinguished. Therefore, the present findings support the potential of *T. nudatum*-mediated Ag-FeO NPs as sustainable bioactive nanomaterials with demonstrated antimicrobial activity and preliminary cytotoxic effects that warrant further investigation.

Future studies should focus on elucidating the molecular mechanisms underlying the antimicrobial and cytotoxic effects of Ag-FeO NPs, including their effects on microbial cell integrity, oxidative stress, and cancer cell signaling pathways. Direct comparison of the *T. nudatum* seed extract and Ag-FeO NPs under identical MTT assay conditions is needed to determine whether nanoparticle synthesis modifies or enhances the cytotoxic activity of the seed extract. Quantitative antimicrobial investigations are also warranted to further validate their biological potential. In addition, improving nanoparticle colloidal stability, assessing long-term biocompatibility, and conducting *in vivo* efficacy and safety studies will be essential before translational applications can be considered. Furthermore, exploring their potential applications in biomedical, agricultural, and environmental fields may facilitate the development of sustainable nanoparticle-based technologies.

## Data Availability

The original contributions presented in the study are included in the article/supplementary material, further inquiries can be directed to the corresponding author.
